# A novel canine reference genome resolves genomic architecture and uncovers transcript complexity

**DOI:** 10.1038/s42003-021-01698-x

**Published:** 2021-02-10

**Authors:** Chao Wang, Ola Wallerman, Maja-Louise Arendt, Elisabeth Sundström, Åsa Karlsson, Jessika Nordin, Suvi Mäkeläinen, Gerli Rosengren Pielberg, Jeanette Hanson, Åsa Ohlsson, Sara Saellström, Henrik Rönnberg, Ingrid Ljungvall, Jens Häggström, Tomas F. Bergström, Åke Hedhammar, Jennifer R. S. Meadows, Kerstin Lindblad-Toh

**Affiliations:** 1 Science for Life Laboratory, Department of Medical Biochemistry and Microbiology, Uppsala University, Uppsala, Sweden; 2 Department of Veterinary Clinical Sciences, University of Copenhagen, Frederiksberg D, Denmark; 3 Department of Animal Breeding and Genetics, Swedish University of Agricultural Sciences, Uppsala, Sweden; 4 Department of Clinical Sciences, Swedish University of Agricultural Sciences, Uppsala, Sweden; 5 Broad Institute of MIT and Harvard, Cambridge, MA USA

**Keywords:** Genomics, Genome assembly algorithms

## Abstract

We present GSD_1.0, a high-quality domestic dog reference genome with chromosome length scaffolds and contiguity increased 55-fold over CanFam3.1. Annotation with generated and existing long and short read RNA-seq, miRNA-seq and ATAC-seq, revealed that 32.1% of lifted over CanFam3.1 gaps harboured previously hidden functional elements, including promoters, genes and miRNAs in GSD_1.0. A catalogue of canine “dark” regions was made to facilitate mapping rescue. Alignment in these regions is difficult, but we demonstrate that they harbour trait-associated variation. Key genomic regions were completed, including the Dog Leucocyte Antigen (DLA), T Cell Receptor (TCR) and 366 COSMIC cancer genes. 10x linked-read sequencing of 27 dogs (19 breeds) uncovered 22.1 million SNPs, indels and larger structural variants. Subsequent intersection with protein coding genes showed that 1.4% of these could directly influence gene products, and so provide a source of normal or aberrant phenotypic modifications.

## Abstract (web summary)

Here, the authors report an improved de novo reference genome for the domestic dog based on a female German Shepherd named Mischka. The new genome increases contiguity 55-fold over the previous dog assembly and uncovers functional elements that were not previously identifiable.

## Introduction

Domestic dogs have lived alongside humans for at least 10,000 years^1,2^, and during this time, they have adapted to a shared environment and diet, while being selectively bred for traits such as morphology^3^ and behaviour^4^. Humans and dogs also share orthologous genes, genomic architecture and disease sets, placing the dog as an important comparative species for human genetics and genomics. Taking advantage of pet dog medical records, within breed homogeneity and disease risk enrichment, it has been possible to provide insights into both rare and common spontaneous disease. The Online Mendelian Inheritance in Animals website (OMIA, June 2020, omia.org) currently catalogues 774 canine traits with linked genetic associations, 234 of which are likely causative in the canine models for human disease. The types of canine variants implicated in disease range from single-nucleotide polymorphisms (SNPs) (e.g. a missense variation in *SOD1* leading to degenerative myelopathy^5^) through complex genomic rearrangements (e.g. a deletion in the repetitive interferon alpha gene cluster associated with hypothyroidism^6^), and were identified with canine SNP chips, e.g., CanineHD BeadChip (Illumina), genotyping complemented with imputation^7^ or genome and transcriptome sequencing of individuals, families^8^ or large populations^3^. Clearly, genome contiguity as well as gene and regulatory element annotation from a range of diverse breeds and tissues are all required to translate association to causation.

The current canine reference genome, CanFam3.1, is based on a 2005 7.4× Sanger sequencing framework^9^, improved in 2014 with multiple methods to better resolve euchromatic regions and annotate transcripts from gross tissues^10^. However, it still contains 23,876 gaps, with 19.6% of these within gene bodies, and a further 9.8% located a mere 5 kb upstream of predicted gene start sites. These gaps result from the accumulation of regions that are difficult to sequence, and are in part due to the loss of *PRDM9* which leads to genomic sections with very high GC content^11^. The consequence of this is the loss of promoters, CpG islands and other regulatory elements from the reference; sequences which may hold the key to deciphering complex traits^12,13^.

To drive canine comparative genomics forward, we generated a high-quality canine reference assembly using a combination of Pacific Biosciences (PacBio) long read sequencing, 10x Genomics Chromium Linked Reads (henceforth called 10x) and HiC proximity ligation. The new reference, UU_CFam_GSD_1.0/canFam4 (henceforth called GSD_1.0), was subsequently annotated with both novel and published whole-genome sequencing (WGS), assay for transposase-accessible chromatin (ATAC) and RNA sequencing to enhance gene models and variant annotation. A liftover of gap regions from CanFam3.1 showed 23,251/23,836 elements contain uniquely anchored sequences in GSD_1.0, and annotation of the new reference resulted in 159 thousand transcripts across 29,583 genes. This novel data open the door to the identification of functional variants underlying complex traits, especially in difficult to sequence, and often biologically important regions.

## Results and discussion

### De novo assembly

**Fig. 1:**
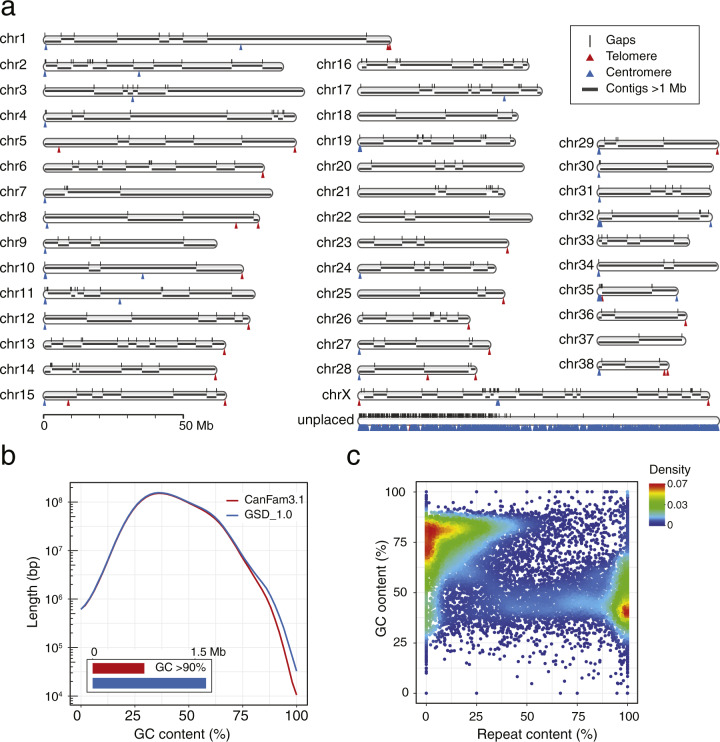
Features of the novel canine assembly. **a** GSD_1.0 ideogram showing chromosomes, contigs, gaps, centromere and telomere repeats. All unplaced sequences were concatenated into a single scaffold (segmental duplications, 58.1%; centromeric repeats, 30.1%). **b** Comparison of GC content (50 bp window) between GSD_1.0 and CanFam3.1. **c** Sequence characteristics of filled CanFam3.1 gaps in GSD_1.0. These are predominately high in GC or repeat content.

Mischka, a 12-year-old female German Shepherd, was selected as the source for our high-quality reference genome assembly. Mischka was free of known genetic disorders, and when compared with additional German Shepherd sourced from within Sweden, was found to be genetically representative of the breed (Supplementary Fig. 1). We sequenced the genome using ~100× coverage PacBio long reads and assembled these in contigs with the standard FALCON method^14^. Further scaffolding using 94× of 10x and 48× of HiC linked reads resulted in 39 single-scaffold chromosomes (total 2.35 Gb) and 2159 unplaced scaffolds (total 128.5 Mb; Fig. 1a). The latter contigs predominantly contain segmental duplications (58.1%) and centromeric repeats (30.1%; Supplementary Fig. 2).

### Reference benchmarking

Compared to CanFam3.1, the contiguity of GSD_1.0 has been improved 55-fold, reaching a contig N50 of 14.8 Mb (Supplementary Fig. 3), with only 367 gaps in the chromosome (chr) scaffolds (Table 1 and Fig. 1a). The identified sequence with extreme GC content (>90% in 50 bp windows) increased from 0.8 to 1.7 Mb (Fig. 1b), leading to a 14% increase in the average length of CpG islands (1056 vs 926 bp, *P* = 8.4 × 10^−4^, *t*-test). Meanwhile, we examined the CanFam3.1 gaps that could be considered closed (23,251/23,836 gap elements from CanFam3.1 have sequence in GSD_1.0; see “Methods”), and found that these regions have either high GC or high repeat content (Fig. 1c).

**Table 1:** Assembly statistics of GSD_1.0 compared to CanFam3.1.

	GSD_1.0	CanFam3.1
Number of contigs	2783	27,104
N50 (L50) contig	14,840,767 bp (57)	267,478 bp (2436)
Number of scaffolds	2198	3268
N50 (L50) scaffolds	64,299,765 bp (15)	63,241,923 bp (15)
Number of Gaps	585	23,876
Gap density (gaps/Mb)	0.24	9.9
Total bases	2,482,000,080 bp	2,410,976,875 bp
Total ungapped bases	2,481,941,580 bp	2,392,715,236 bp

### Repeat structure

Approximately 42.7% of the genome is repetitive sequence, with the three major categories being LINEs (504 Mb), SINEs (253 Mb) and LTRs (120 Mb) (Supplementary Fig. 4 and Supplementary Table 1). Long read technology allowed for the further resolution of centromeric repeats, and based on their positions, the orientation of chr 27 and 32 were reversed compared to CanFam3.1. These two chromosomal re-orientations were further supported by published recombination rate patterns and fluorescence in situ hybridization experiments^15^. In addition, the q-arms of 21 autosomes now begin with centromeric repeats, and 17 autosomes end in telomeric repeats (Fig. 1a). As expected, the sub-metacentric chr X has telomeric repeats at each end, and a clear centromeric signal at 49.4–49.9 Mb. Throughout the genome we found 10 internal centromeric and 7 internal telomeric repeats. These may indicate ancient centromere and telomere positions prior to chromosomal rearrangements and most were also present in the previous reference genome assembly.

### Functional annotation

To resolve transcript complexity and account for the CanFam3.1 gap closures in GSD_1.0, we generated more than 70 M nanopore and PacBio full-length cDNA reads from 40 tissues (including 15 brain regions; Supplementary Table 2), and combined this with 24 billion public RNA-seq paired reads (Supplementary Data 1). The annotation consisted of 159 thousand transcripts in 29,583 genes; of which 20,654 had an open reading frame (ORF) of at least 100 amino acids and 19,691 genes had a significant BLAST hit against proteins in Swissprot or ENSEMBL. Further, 7725 were defined as long noncoding genes. Compared to proteins extracted from CanFam3.1, our new GSD1.0 annotation has a higher number of genes with BLAST hits and the number of genes with a full-length match has increased by 11% (Supplementary Fig. 5). Gene predictions and non-dog refSeq alignments were used to identify potentially missed genes that did not overlap with our annotation, yielding an additional 874 protein-coding genes with BLAST evidence. Using a combination of new miRNA-seq reads and public data we identified a conservative set of 719 miRNAs, similar to the set found for CanFam3.1^16^. Among the novel miRNAs, a copy of the highly expressed Mirlet-7i was identified in a filled CanFam3.1 gap region (Supplementary Fig. 6). This miRNA has been implicated in several human diseases, including multiple sclerosis^17^, gastric cancer^18^ and breast cancer^19^, but has yet to be extensively studied in dogs.

**Fig. 2:**
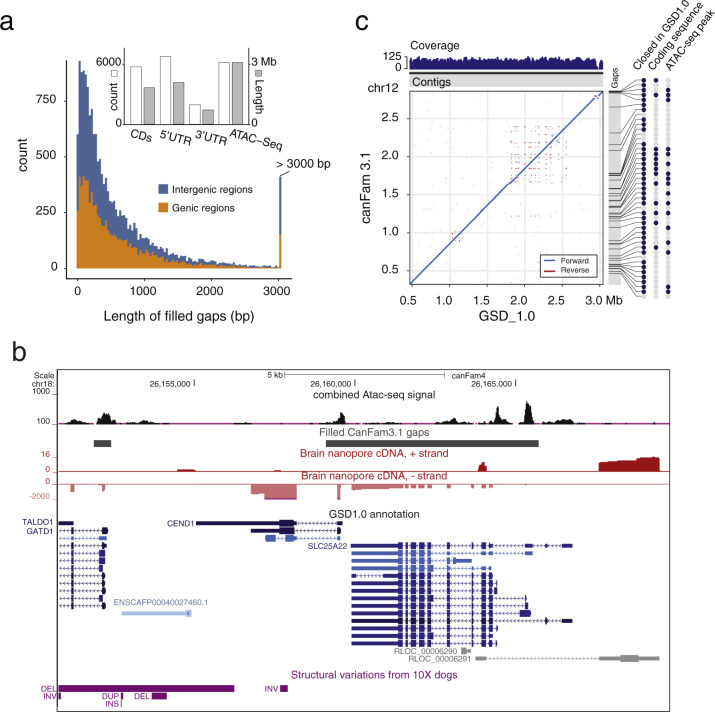
Filled CanFam3.1 gaps and functionality. **a** Size distribution and overlap with exons and promoters for the filled CanFam3.1 gaps. **b** Representative GSD_1.0 annotation from the UCSC track hub highlighting available data and an example of a gene hidden in CanFam3.1. **c** Sequence comparison of DLA on chr 12 between CanFam3.1 and GSD_1.0. The blue indicates a forward alignment and the red indicates a reverse alignment.

We identified 7468 closed CanFam3.1 gaps containing either an exon or promoter sequence as defined by ATAC-seq peaks, accounting for 5743 unique coding exons which were missing in CanFam3.1 (Fig. 2a). Notably, eight genes with expression across multiple tissues were completely absent or represented by pseudogenes in CanFam3.1 but were now available for interrogation (*PSMA4, CDHR5, SCT, PAOX, UTF1, EFNA2, GPX4* and *SLC25A22*). These genes have diverse functions ranging from embryonic stem cell co-activator (*UTF1*) to osmoregulation (*SCT*). Both *CDHR5* and *SLC25A22* (Fig. 2b) have been investigated as biomarkers for either renal^20^ or colorectal^21^ cancers.

### Implications for research

We assessed the chromosomal order and contiguity of regions essential to the study of cancer and immunological disease. Using the human COSMIC^22^ gene list as a baseline, we affirmed that 282 tier1 and 78 tier2 genes are now completely captured, including *HOXD13* and *KLF4* (Supplementary Table 3). Both have been implicated in human breast cancer; *HOXD13* methylation status functions as a prognostic indicator^23^ and deubiquitination of KLF4 promotes metastasis^24^ (Supplementary Fig. 7). Two main dog leucocyte antigen (DLA) regions on chr 12 (Fig. 2c) and 35 (Supplementary Fig. 8a) are contiguous in GSD_1.0^25^ (covering 2.58 and 0.61 Mb, respectively) and contain new coding and potential regulatory sequences absent in CanFam3.1 gaps. Meanwhile, small DLA regions on two other chromsomes^26^ (chr7, 1 kb, *C1PG-26* and chr 18, 3Kb, *DLA-79*) remain contiguous in GSD_1.0. Contiguous sequence was also reported for both the T cell receptor alpha (TRA) and T cell receptor beta (TRB) loci on chr 8 and 16, respectively (Supplementary Fig. 8b, c).

### Comparison to canine assemblies

Four additional canine genome assemblies have recently been deposited in NCBI (Supplementary Table 4). For each assembly, we compared BUSCO^27^ scores and mappability using in-house Iso-Seq cDNA alignments generated above from a beagle dog (Supplementary Table 2). With GSD_1.0 it was possible to map >5% more bases from 25,609 of Iso-Seq reads compared to CanFam3.1 (4.8% of total reads; Supplementary Fig. 9). This was a higher fraction than for the other assemblies (Supplementary Table 5 and Supplementary Fig. 10). GSD_1.0 had the second highest BUSCO score for complete genes (95.5%), but each canine assembly is of value to the community and may serve different experimental goals.

### Genome variation

**Fig. 3:**
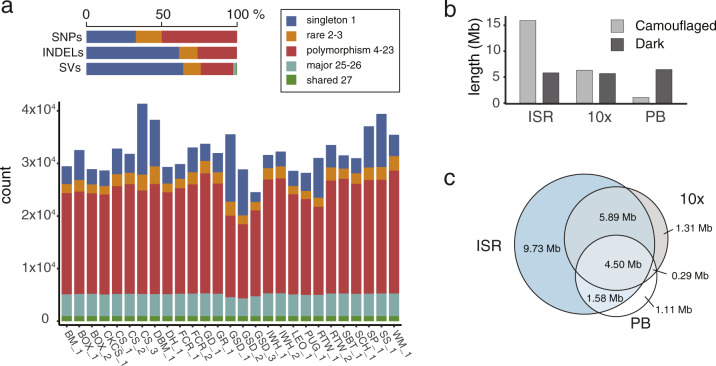
Genome variation and dark/camouflaged regions. **a** SNPs, indels and structural variations shared among Mischka and the 27 10x sequenced dogs. **b** The total length of dark and camouflaged regions detected from Illumina short reads (ISRs), 10x and PacBio sequencing. **c** Intersection of merged dark and camouflaged regions from different datasets.

Polymorphisms detected in 27 dogs (19 breeds) were extracted from 10x sequencing data to facilitate the investigation of genome features and across-breed variant segregation (Supplementary Table 6). We identified 14,953,199 SNPs, 6,958,645 indels and 217,951 structural variants (SV, average 2.4 kb; Fig. 3a). Of these, 42.1% were private, 57.9% polymorphic across multiple individuals and 1.4% overlapped with protein-coding regions (295,112 SNPs and 16,654 SVs). Intersection with existing SV catalogues based on either SNP or aCGH arrays^28–30^ showed between 12.6 and 39.0% agreement, but these numbers are likely a reflection of within project breed and detection technology. 10x sequencing allowed for the detection of many novel SVs with small to medium size (≥30 kb) with accurate breakpoints.

### Genome “dark” regions unmasked

The majority of publicly available dog WGSs were generated with short read technologies. To facilitate the reanalysis of these resources with GSD_1.0 we aimed to identify the genome’s “dark” regions^31^; those sections either not adequately covered due to sequencing method (dark by depth, dark) or to which unique alignment is not possible (camouflaged regions, camouflaged). We defined GSD_1.0 dark and camouflaged regions for Illumina short reads (ISRs), 10x, and PacBio (PB) sequencing (see “Methods”). Dark regions comprised 5.8, 5.7 and 6.4 Mb, respectively, while camouflaged regions comprised 15.9, 6.4 and 1.0 Mb (Fig. 3b). Intersection showed that while 10x could rescue 11.3 Mb dark and camouflaged regions not seen with ISR (9.73 + 1.56 Mb), more than half of this again (5.9 Mb) could be further recovered by PacBio (Fig. 3c). We noted six tier1 & 2 COSMIC genes that contained either dark or camouflaged regions (*EPHA3*, *RALGDS*, *LRP1B*, *CSMD3*, *ZMYM2*, *PTEN*; 0.8–6.6% of coding region hidden), potentially masking drivers of disease. Due to the nature of dark and camouflaged regions, default practices will not allow for the mapping of ISR reads to, and subsequent variant extraction from, these positions. Instead, we extracted variants overlapping annotated dark and camouflaged regions from our “healthy” 10x dataset, and in doing so, identified 51,994 SNPs and indels, including 19,340 intronic and 2074 exonic variants. Many of these variants were embedded in genes that may be important for morphology or associated with disease. For example, 14 variants were found within seven intronic *TYRP1* ISR dark/camouflaged regions (Supplementary Fig. 11a): a gene linked to brown colour in dogs^32^ and melanoma in humans^33,34^. Likewise, 76 variants were found in *ADCY2* ISR dark/camouflaged regions (Supplementary Fig. 11b). Polymorphisms in this gene have previously been associated with psychiatric and neurological disorders (bipolar disorder^35^ and Alzheimer’s disease^36^), and response to associated drug therapies of schizophrenia^37^ in humans.

### Chromosome mis-assembly resolved

**Fig. 4:**
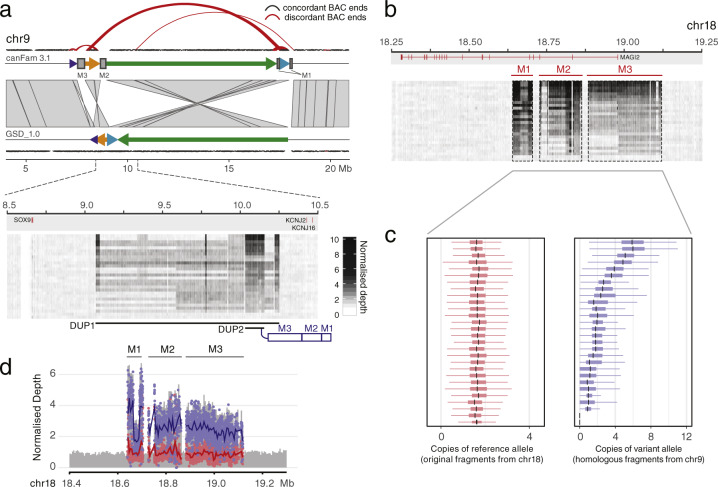
Correction of an inverted region in chromosome 9. **a** Four fragments from the region were rearranged in GSD_1.0. The order was further confirmed using CanFam3.1 BAC clone (CH82) end sequences. Forty-nine discordant end pairs (red curves; >500 kb or not in a forward–reverse direction) were found at the edge of rearranged fragments in CanFam3.1, whereas these were properly mapped in GSD_1.0. From this region, three homologous chr 18 fragments spanning *MAGI2* (M1, M2 and M3) were present on chr 9 of CanFam3.1, but missing in the GSD_1.0. We proposed that those homologous fragments should be located together with a duplication (DUP2, chr 9: 10.03–10.16 Mb) within a large duplicated region (DUP1, chr 9: 9.07–10.25 Mb). **b** Reads from both original and homologous M1, M2 and M3 fragments were mapped to chr 18 of GSD_1.0. **c** Mischka and all 10x dogs have only two original chr 18 copies M1, M2 and M3, but carry between 0 and 6 copies of the chr 9 homologous fragments. **d** The example plot of normalised depth illustrates how the copy number of the reference alleles and variant alleles were measured to distinguish the original (red) and homologous (blue) of M1, M2 and M3.

A direct comparison of CanFam3.1 and GSD_1.0 revealed a complex ~10 Mb inverted region on chr 9 that harboured *SOX9* and was previously implicated in canine XX disorder of sex development (DSD)^38–40^. Three polymorphic regions homologous to parts of *MAGI2* on chr 18 (M1, M2, M3) have been inserted upstream of *SOX9* (Fig. 4a, b). In DSD, having multiple copies of a copy number variation (CNV) overlapping M2^39^ was shown to be associated with altered SOX9 function during gonadal development. Using HiC and BAC end sequencing data, we confirmed that the inverted GSD_1.0 orientation was correct and refined the placement of regions M1, M2 and M3 (Fig. 4a). These chr 9 insertions are missing from GSD_1.0, but allelic depth analysis revealed that most 10x dogs (26/27) carry between 2 and 6 chr 9 copies (Fig. 4c, d), similar to the estimates reported for non-DSD dogs^40^. Recently it was shown that the DSD phenotype presents in a breed-specific manner, and is influenced by the combination of an SNP and CNVs in this region^38,40^. However, as this inversion contains numerous genes and regulatory elements, this rearrangement, including multiple CNV expansions, has the potential to impact additional canine traits.

### CYP1A2 locus variation

**Fig. 5:**
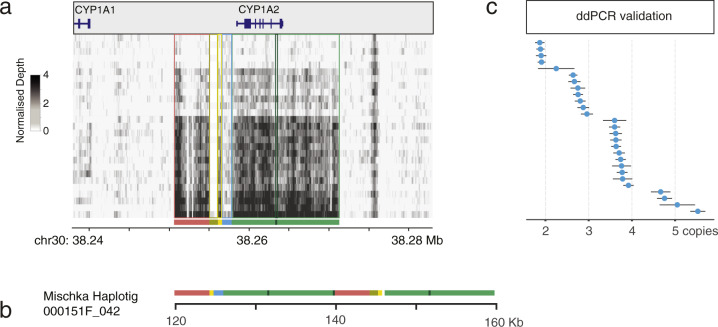
Copy number expansion encompassing CYP1A2. **a** A duplication identified on chr 30 consists of six segments and contains the *CYP1A2* gene. **b** The individual pieces from the reference are plotted as they appear in the alternative haplotig sequence (000151F_042) for Mischka (CNV = 3). Sequence was extracted from the FALCON assembly. Full-length *CYP1A2* sits within copies of the green fragment. **c** The duplication was validated in the 10x sequenced individuals using ddPCR.

To further investigate the impact of SVs on coding genes, we examined the 16.2 kb copy number locus which encompassed *CYP1A2* (Fig. 5a, b). Dogs are used as comparative models for human xenobiotic metabolism, and while a *CYP1A2* premature stop codon (rs852922442 C>T) has been reported^41,42^, the CNV locus expansion has not. The homozygous T genotype can be found in multiple breeds^43^ and results in an array of pharmacokinetic effects, including reduced hepatic drug metabolism^44^. The T allele was observed in 4/27 10x dogs, but in heterozygous form and not segregating with CNV count (2–5 copies; Fig. 5c). Differential gene expression analyses for this and neighbouring genes outside the locus were performed using either liver or spleen tissue from additional individuals (Supplementary Data 2 and Supplementary Table 2). After accounting for *CYP1A2* SNP rs852922442-T, no significant relative gene expression difference was observed, leaving the phenotypic consequence of this expansion unresolved (CNV 3 vs >3; Supplementary Table 7). It may be that the effect in this region is subtle, and so not detectable with qPCR; however, *CYP1A2* is an inducible gene and so the true outcome may only be observed after a drug challenge^45^.

### Conclusion

Through the combination of sequencing technologies, PacBio (~100X) long read, 10x and HiC proximity ligation, we have generated a contiguous, chromosome length scaffolded GSD_1.0 canine reference genome. GSD_1.0 has a 55-fold increased contiguity compared with its predecessor CanFam3.1. This brings the canine reference genome quality in line with other key mammalian species, e.g. human^46^, mouse^47^, and gorilla^48^. For both human and mouse projects, the de novo sequence assembly of multiple individuals from different population backgrounds has revealed novel sequence not found in the single (hybrid in the case of human) species reference, and facilitated the search for population-specific variants which likely contribute to traits of interest, including within the highly polymorphic immune gene clusters^46,47^. While this type of de novo collection is on-going within the canine community, GSD_1.0 is the first genome of reference quality that is further annotated with novel long read RNA sequencing data, allowing for the resolution of transcript complexity through regions with high GC context, or “dark” regions^31^.

The resolution and placement of repeats in GSD_1.0, including non-LTR retrotransposons, will facilitate the study of gene and genome evolution and the process of neofunctionalization across mammalian lineages to an extent not possible previously. Over more recent timespans, these mobile elements can allow for genome slippage, and to the accumulation of within and across population SVs. In human clinical genomics, SVs spanning coding and/or noncoding sequence have been responsible for a range of maladies including cardiac anomalies (OMIM 192430) and intellectual delay and autism (OMIM 608 636). Accordingly, this source of variation is of keen interest in canine genetics, and should facilitate similar lines of investigation. The technology used to read across repeats was also successful in reading into regions of constitutive heterochromatin, allowing for the correction of chromosomal direction (chr 27 and 32) and revealing novel centromeric and telomeric sequences.

Perhaps the largest gain offered by the contiguity of GSD_1.0 is to the accelerating field of low pass genotyping and imputation for trait mapping^7^. The completion of key regions to the investigation of immunological disease and cancer, e.g. DLA and TCR, when combined with large reference populations, will facilitate the more accurate genotyping of these regions and hopefully fast track the process from association to causation. We believe that the catalogues generated here (extended gene models, dark/camouflaged regions, within and across-breed variation), based on the GSD_1.0 framework, will propel the comparison of canine and human genetic disease forward by leaps and bounds.

## Methods

### Reference individual

Mischka, a 12-year-old female German Shepherd, was born and raised in Sweden with known ancestral background and no medical history of genetic disease. Mischka was genotyped with the CanineHD BeadChip (Illumina) and compared to a population of 260 German Shepherds from a previous study^49^. Mischka was assessed to be representative of the population via expected inbreeding value (*F* = 0.037) and multiple dimensional scaling genetic distance measures (PLINK v1.9) and selected for the genome assembly. High molecular weight (HMW) DNA was extracted from blood with MagAttract HMW DNA Kit (Qiagen).

### Genome sequencing

The assembly used multiple sequencing technologies. Long read libraries were prepared with SMRTbell Template Prep Kit 1.0 and 70 SMRT cells were sequenced on the PacBio Sequel system with v2.1 chemistry (Pacific Biosciences; 276.86 Gb data). Linked reads were sequenced from HMW DNA with Chromium libraries (10x Genomics) on an Illumina HiSeq X (2 × 150 bp; 269.75 Gb of data). Dovetail Genomics prepared three HiC libraries which were sequenced on an Illumina HiSeq X (2 × 150 bp paired-end reads; 121.47 Gb data, Supplementary Table 8).

### Assembly construction

De novo assembly used PacBio subreads (>8 kb) with the standard FALCON^50^ v0.5.0 method. After Arrow^50^ (v2.3.3) polishing, the assembly yielded 3656 contigs with an N50 and mean length of 4.66 Mb and 677 kb, respectively. ARCS^51^ v1.05 and LINKS^52^ v1.8.6, with the recommended link ratio (-a) 0.9, were used to scaffold contigs with 10x reads. In all, 1170 FALCON contigs were joined in this step, increasing the scaffold N50 to 18.5 Mb.

### Conflict resolution

Scaffolding correctness was evaluated by aligning scaffold sequences onto the high-density canine linkage map^15^. In all, 21,278 of 22,362 markers (95%) were unambiguously mapped to the assembly by BLAT^53^ v36. Synteny of genetic and physical location of markers was further compared with Chromonomer^54^ v1.0, which showed 207 scaffolds were anchored correctly, but that four had conflicting markers. These four scaffolds were split after careful sequence review confirmed that each discrepancy arose from incorrect inter-chromosomal joining.

### Gap filling and assembly polishing

PBjelly from PBSuite^55^ v15.8.24 was used with PacBio subreads to close 648 gaps. An initial QC scan showed no putative wrong joins, and so long-distance interaction information from HiC (HiRise, Dovetail Genomics) was used to successfully extend scaffolds to chromosome level (scaffold N50: 64.3 Mb). These results were evaluated with the JUICER^56^ pipeline; HiC reads were mapped back to the HiRise assembly and HiC map with intra- and inter-chromosomal interactions visualised. We identified and manually adjusted contigs placed in either the wrong order or orientation (chr 6, 14, 17, 26 and X), and joined separated contigs from the same chromosome (chr 8 and 18). A second round of PBjelly gap filling closed another 110 gaps. The assembly was polished with Arrow (PacBio subreads) and Pilon^57^ v1.22(10x Genomics reads, BWA^58^ v0.7.15 mem mapping). A FreeBayes-based method was applied to further correct indel errors^59^. SNPs and indels were called from short reads aligned to the polished assembly (FreeBayes^60^ v1.1.0). The reference base was replaced with the variant allele at 149,264 positions where 10x sequencing depth was at least 30× and the variant allele ratio was >90% using FastaAlternateReferenceMaker from GATK^61^ v4.1.1.0. A final round of Pilon short read polishing was completed prior to the removal of 68 unplaced contigs with suspected bacterial contamination (Kraken2^62^ v2.0.8).

The correctness of a large rearranged region on chr 9 of GSD1.0 was confirmed through comparison to end sequences from original CanFam BAC clones (CH82 library; NCBI TraceDB). BAC sequences were mapped as paired reads (BWA^58^ mem default setting), to GSD_1.0 and CanFam3.1. End pairs that mapped to both assemblies were compared and defined as concordant when they aligned in forward and reverse direction with a distance <500 kb.

### GC content and repetitive elements

GC content (%) was assessed in 50 bp windows (NUC from BEDTools^63^ v2.29.2). CpG islands were detected with the “cpg_lh” script from UCSC utilities (http://hgdownload.soe.ucsc.edu/admin/exe/linux.x86_64.v369/), a modified method from Gardiner-Garden^64^. The unique mappability of GSD_1.0 was tested with different k-mers (50/150/250 bp in GEM-Tools^65^ v1.71). Repetitive elements were annotated by Repeat Masker v4.0.8 in a sensitive mode (http://www.repeatmasker.org) with a combined library (dc20171107-rb20181026). Telomere repeats, “TTAGGG”, were highlighted on both strands with fuzznuc (EMBOSS^66^ v6.6.0). Putative telomere sequences were defined as at least 12 consecutive repeats with less than 11 variant bases between each, and multiple sequences were merged if within 100 bp. Centromeric regions were defined based on satellite repeat^67^ (CarSat1/Carsat2/SAT1_CF) content in 5 kb windows. Putative centromere sequences were annotated if the repeat content was >80%.

### RNA preparation and long read cDNA sequencing

Multiple RNA samples from Beagles were used for RNA sequencing (Supplementary Table 2). First, total RNA from hypothalamus (RIN > 8; Zyagen) was purchased for sequencing via PacBio Iso-Seq express protocol. Two libraries were run on two separate SMRT cells using the Sequel system, and yielded ~500,000 reads each with mean read lengths of 2452 and 451 bp. Total RNA from a further 24 tissues (including 15 brain regions; Supplementary Table 2) was extracted using a standard TRIzol protocol (Invitrogen) and used for nanopore cDNA and Illumina miRNA-sequencing. The PCR strand-switch protocol and the SQK-LSK109 kit were used for MinION sequencing (Nanopore). All tissue samples were amplified with PBC096 barcoding for 8–10 cycles with both LongAmp (female samples, 62 °C annealing; NEB) and PrimeSTAR GXL (both sexes, 64°C annealing; Takara Bio), with a 10 minutes extension time. The retina sample was sequenced using both the nanopore direct cDNA sequencing kit SQK-DCS109 and as stranded 2 × 150 bp reads on a NovaSeq 6000 S4 lane (Illumina). Reads were base called with the high accuracy model in guppy (v3.6 for direct cDNA and v3.3 for amplified samples). Qcat and pychopper (https://github.com/nanoporetech/) were used to demultiplexed reads and to identify and orient fully sequenced reads. Mapping accuracy was increased by only using reads with a quality value above 15. For PacBio, full-length circular consensus sequencing (CCS) reads with at least three passes were selected. The long read cDNA runs were mapped with Minimap2^68^ (v2.17) with the options -x splice -G 500000 and --junc-bed with splice junctions identified from the Illumina alignments. These settings improved mapping both to genes with long introns and to short exons. MicroRNA libraries were made with the NEXTFLEX small RNA library kit v3 (PerkinElmer) and 25 million reads were generated with a NextSeq500 instrument (75 bp high-output kit v2.5 in paired-end mode; Illumina).

### Gene annotation

Public Illumina stranded RNA-seq runs with paired reads of at least 100 bp were downloaded from NCBI using the SRA-Explorer (https://sra-explorer.info/). Samples were selected to cover a diverse set of dog tissues and breeds (Supplementary Data 1). Reads from the same study and tissue were combined and adaptors were trimmed with BBmap. HISAT2^66^ and RSeQC^69^ were used on a small subset of reads for each sample to infer library type. Stringtie2^67^ superreads module was used to assemble and merge transcripts from Illumina reads, with setting -f 0.05 as the threshold for isoform expression. Stringtie2 assemblies were made both for individual samples and with combined samples from the same tissue type. PacBio iso-seq alignments were combined with alignments of nanopore full-length cDNA reads for assembly with Stringtie2 with options “-L -c 3 -s 10 -f 0.05” to suppress low-coverage transcript models from internal priming and partially spliced mRNAs. Stringtie2 was further used to merge transcripts from the individual assemblies of long and short reads. Assembled transcripts were processed with TAMA tools^68^ for ORF detection and BLAST parsing to identify coding regions based on hits against a database of curated proteins from Uniprot_Swissprot and proteins from the latest ENSEMBL dog annotation (v100, Great Dane assembly). The id of the protein was determined from the longest BLAST hit from the top five hits with an *E*-value below 10^−10^. We found the Stringtie assembly sometimes missed low-coverage genes that were close to, but not overlapping, highly expressed genes. To make the assembly as complete as possible we therefore combined the output from multiple runs, used TAMA to assemble long reads not overlapping with Stringtie2 transcripts and included novel transcripts if they were multi-exonic and had a blast hit covering at least 50% of the target. Additional filtering was applied to remove transcripts that, (1) were long single exon transcripts (>10 kb and <10% intronic sequence) or (2) originated from genomic polyA/T regions. Gffread^70^ was used to re-group transcripts into genes, retaining only one transcript per unique CDS region. Finally, transcripts which had either >2 exons downstream of the stop codon, or a bad BLAST classification (<50% hit) were removed if they belonged to a group with high scoring transcripts (Supplementary Fig. 12). Long noncoding genes were defined as having at least two exons, a length of >200 bases, no ORF longer than 100 amino acids and no overlap with protein-coding exons on the same strand.

### miRNA identification

Public microRNA-seq samples (Supplementary Data 1) were combined with the above brain microRNA-seq reads (Total reads, 1.3 billion). Reads were included if they were between 20 and 30 bases after adaptor trimming. Bowtie alignments of unique sequences were used for MiRDeep2^71^ analysis and compared to known dog and human miRNAs (miRBase) in order to identify the position of both known and novel miRNAs.

### ATAC-seq analysis

Reads from BARKbase^72^ (Supplementary Data 1) were aligned with BWA mem and peaks called with Genrich (https://github.com/jsh58/Genrich). BedGraph files were produced with BEDTools.

### CanFam3.1 gap comparison

CanFam3.1 gaps were defined as any continuous ambiguous”N” bases, and for each, 1 kb flanking sequences were extracted and mapped as pairs to GSD_1.0 (BWA mem). CanFam3.1 gaps were considered closed when (1) flanking sequence pairs could be mapped properly in the same scaffold with mapping quality >20; (2) the distance between pairs was less than 100 kb; and (3) no GSD_1.0 gap was present in the sequence between pairs. This approach identified the sequence for 18,649 of 19,553 (95.4%) gaps from assembled chromosomes, and 1563 of 4323 (36.2%) gaps from unplaced scaffolds of CanFam3.1 in GSD_1.0. The flanking sequences of 3072 gaps overlapped each other in GSD_1.0, suggesting artificial gaps in CanFam3.1 that can be considered closed in GSD_1.0. For the other closed gaps, we extracted the filled sequences from GSD_1.0 and calculated GC and repeat content. BEDTools was used to intersect exons, miRNA and ATAC-seq peaks mapped above with filled CanFam3.1 gaps. Specifically, we looked for novel genes from the filled CanFam3.1 gaps. A novel gene was defined if it (1) had at least 80% of the gene body identified from the filled CanFam3.1 gaps; (2) was not a pseudogene; (3) had not been annotated in the unplaced scaffolds of CanFam3.1; and (4) did not have the duplicated/homologous fragment in another region of the genome. With these thresholds, we found eight novel genes from the filled CanFam3.1 gaps, and all located in regions with good synteny of human hg38 assembly.

### Region comparison

We compared dog DLA, TRA and TRB regions between GSD_1.0 and CanFam3.1 by NUCMER^73^. By lifting the human major histocompatibility complex regions from the genome reference consortium, two main DLA regions were found in GSD_1.0: chr 12: 0.45–3.05 Mb (*TRIM39* → *SYNGAP1*), chr35: 27.0–27.9 Mb (*GPX6* → *TRIM26* gene). Two additional DLA regions, chr7:59.69 Mb (1 kb, *C1PG-26*) and chr 18: 41.56 Mb (3 kb, *DLA-79*) were identified by the previous study^26^.

### Assembly benchmark with Busco and Iso-Seq data

BUSCO^27^ v3.0.2b was run with the mammalia_odb9 dataset. Mappability was assessed with Iso-Seq data using only PacBio CCS reads supported by >10 subreads (483,702 reads). CCS reads were mapped with minimap2 v2.17, and the percentage of mapped bases per read calculated according to the “difference string” in cs tag. With these methods, GSD_1.0, CanFam3.1 and four newly released canine assemblies, Luka (Basenji), Nala^74^ (German Shepherd), Zoey^75^ (Great Dane) and Scarlet^76,77^ (Golden Retriever, Supplementary Table 4).

### 10x and standard ISR mapping

HMW DNA was extracted from the blood of 27 additional dogs (19 breeds), and Chromium library preparation and sequencing completed as per “Genome sequencing”. Sequencing depth ranged between 30 and 93× (Supplementary Table 6). Unplaced GSD_1.0 scaffolds were concatenated into a single scaffold with 500 “N” base spacers and 10x reads were mapped to each with the Long Ranger v2.2.2 WGS pipeline (10x Genomics). 10x breed-matched ISR data were downloaded for 25 individuals (Supplementary Table 9) and mapped to GSD_1.0 (BWA mem, default settings). SNPs and short indels were detected in 10x and ISR dataset using appropriate modules from GATK4. Variants were called from alignment by HaplotypeCaller, and further merged by the CombineGVCFs and GentoypesGVCFs. The SNPs and indels were filtered by SelectVariants with “QD < 2.0| | FS > 60.0| | MQ < 40.0| | MQRankSum < −12.5| | ReadPosRankSum < −8.0” and “QD < 2.0| | FS > 200.0| | ReadPosRankSum < −20.0”, respectively.

### Dark and camouflaged region detection

Both depth and mapping quality were calculated for each sample in each 10x or ISR dataset. For sequencing coverage, bamCoverage (Deeptools^78^ v3.3.2) with a 25 bp window was used, with unmapped reads and secondary alignments excluded from the analysis. For the same windows, the proportion of reads with mapping quality >10 was also assessed. Regions dark by depth (dark) were defined as windows with coverage ≤5×, with threshold adjusted for sequencing depth. A lower cutoff was applied in low-coverage samples to select a maximum of 60 Mb (Supplementary Data 3). The individual dark regions were merged, and the dark fraction for each window was assessed for both ISR and 10x datasets: windows with *F*_dark_ > 0.9 (90% individuals, in at least 23 ISR dogs or 25 10x dogs) retained as the candidate dark regions. Camouflaged regions (camouflaged) were defined if the coverage was ≥10× and the proportion of high mapping quality reads was less than 10%. We searched for and merged the genomic windows that reached the threshold from each dog. As the camouflaged regions detected in one individual could have been assigned as dark in others, we excluded those dark dogs before we calculated the fraction of camouflaged bases for each window. Any window with *F*_camouflaged_ > 0.9 was selected as a candidate.

### Structural variation (SV) detection

We scanned the genomes of 27 10x dogs using four SV callers. The first, Long Ranger, was used to call the SVs in two size ranges. Medium SVs spanning from 50 to 30 kb were detected by examining the haplotype-specific coverage drops and discordant reads pairs. Larger-scale SVs, >30 kb, were identified as regions where paired coverage of genomic loci shared many more barcodes than expected by chance. Candidate SVs were further refined and categorised (DEL deletion, CNV copy number variant, INV inversion) by comparing the layout of reads and barcodes around the breakpoints. Three additional callers were adapted to discover other types of median size SVs (50 bp–30 kb). GridSS^79^ and Manta^80^ are assembly-based callers which have been reported to have a good performance in different studies^81,82^. Both detected SVs using evidence from split and paired reads, and also assembled the sequences of breakpoints to accurately estimate these positions. The type of SVs called by GridSS was determined by the orientation of reads from the breakpoints using a R script (https://github.com/PapenfussLab/StructuralVariantAnnotation). From the three callers above, only high-quality SV calls marked as “PASS” in vcfs were kept for analysis. Lastly, CNVnator^83^ predicted CNVs by a read-depth (RD) approach. A 150 bp bin size was used for screening, and retained SVs were required to have a *p* value <0.05 for a RD *t*-test statistic (“e-val1”) and the probability of RD frequency <0.05 in a gaussian distribution of (“e-val2”). The result was converted into VCF form using the “cnvnator2VCF.pl” script from the CNVnator package. For each 10x sample, the filtered median SVs from all four callers were merged by the SURVIVOR^84^, and combined with the large size SVs called from Long Ranger. Chr X SVs that were only supported by CNVnator were pruned as the algorithm lacks the right model sex chromosome. SVs were further merged across individuals into a nonredundant SVs set.

### SV validation and genotyping

Four DELs and four CNVs which overlapped protein-coding genes that were polymorphic within the 10x dataset (>3/27 individuals) were selected (Supplementary Data 2). SV breakpoints were confirmed with Sanger sequencing where possible. PCR was performed with either PrimeSTAR GXL DNA Polymerase (Takara) or AmpliTaq Gold DNA Polymerase (Applied Biosystems) according to the manufacturer’s recommendations. PCR fragments were cloned using either Zero Blunt or TOPO TA Cloning Kit (Invitrogen) depending on PCR overhang. Plasmid DNA was extracted using QIAprep Spin Miniprep Kit (Qiagen), PCR products and plasmids sequenced using the Mix2Seq service (Eurofins Genomics) and analysed using CodonCode Aligner v6.0.2 (CodonCode). For *CYP1A2* CNV genotyping, ddPCR absolute quantification (BioRad) was performed and quantified as before^85^. *CYP1A2* C1117T was genotyped according to a published method^86^. New Primers and probes were designed using Primer3 v0.4.0 (http://bioinfo.ut.ee/primer3-0.4.0/) and collated in Supplementary Data 2.

### Gene expression

Total RNA was extracted from liver and spleen tissues using the AllPrep DNA/RNA/miRNA Universal Kit (Qiagen) according to the manufacturer’s specification and including on-column *DNaseI* treatment (Supplementary Data 4). In total, 1000 ng of total RNA was reverse transcribed using the Advantage RT-for-PCR Kit (Takara) and qPCR performed in quadruplet using SYBR Green PCR Master Mix (Thermo Fisher Scientific) and 900 nM primers in a QuantStudio 6 Real-Time system (Thermo Fisher Scientific) with standard cycling and dissociation curve analysis. Two housekeeper primer sets (RPS19 and RPS5) were assessed for stability (*Normfinder*^87^ R package) and used in combination to calculate relative gene expression^88^. These calculations included primer specific efficiencies and used the average Ct from all control samples for initial delta Ct normalisation. wilcox.test in R was used to assess the significance of between genotypic class gene expression changes.

### Statistics and reproducibility

Statistical analysis was performed by R v3.6.0 with algorithms and packages as described.

### Ethics approval and consent to participate

Approval was obtained from dog owners before collecting the biological samples at veterinary clinics. Ethical approvals for sampling were granted by Uppsala Animal Ethical Committee and Swedish Board of Agriculture (C139/9, C2/12, C12/15). Importation of canine tissues was approved by Jordbruksverket (6.7.18-14513/17).

### Reporting summary

Further information on research design is available in the Nature Research Reporting Summary linked to this article.

## Supplementary information

Supplemental Material

Description of Additional Supplementary Files

Supplementary Data 1

Supplementary Data 2

Supplementary Data 3

Supplementary Data 4

Reporting summary

## Data Availability

The PacBio long reads, HiC, and Illumina 10x data of Mischka are available in SRA under BioProject PRJNA587469. The Illumina 10x data of 27 dogs are available in SRA under BioProject PRJNA588624. miRNA & RNA sequencing data are available in SRA under BioProject PRJNA657719. The canFam_GSD_1.0 assembly is deposited in DDBJ/ENA/GenBank under JAAHUQ000000000, and also available in UCSC browser (http://genome-euro.ucsc.edu/cgi-bin/hgTracks?db=canFam4).

## Code availability

Scripts used in the study are available at the GitHub repository (https://github.com/Chao912/Mischka/).
