# Supplementary material for: A novel canine reference genome resolves genomic architecture and uncovers transcript complexity
Source: Commun Biol. 2021 Feb 10;4:185. doi: 10.1038/s42003-021-01698-x (PMC7875987; doi:10.1038/s42003-021-01698-x)
Supplement: Supplementary file 1 — Supplemental Material [file 42003_2021_1698_MOESM1_ESM.pdf]

Supplementary Figures

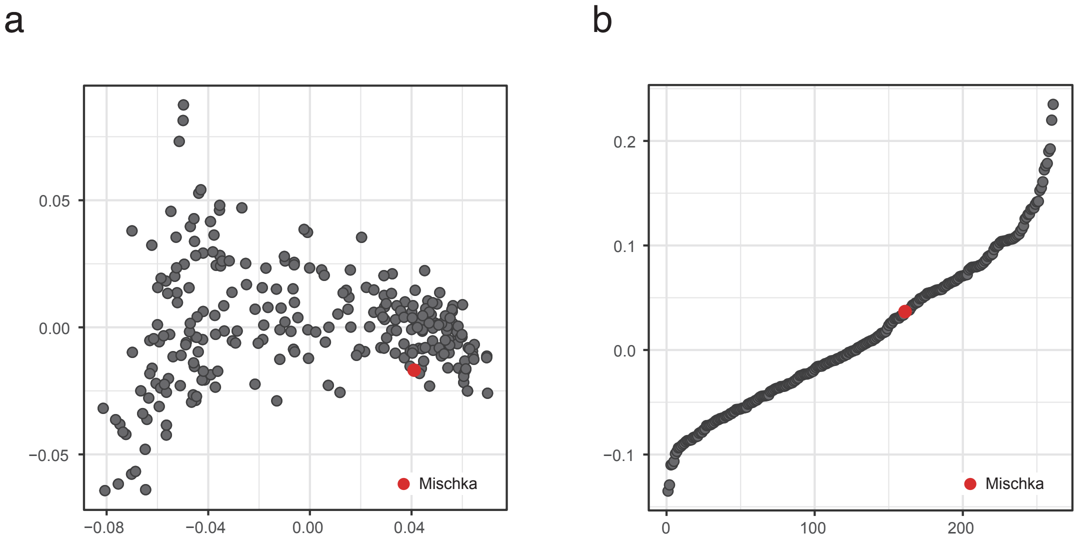

Supplementary Figure 1. Genetic relationship between Mischka and other German Shepherds. a) Multidimensional scaling (MDS) plot shows that Mischka falls within the cluster of 260 German Shepherds from a previous study[1]. b) The inbreeding coefficient for Mischka is 0.037, indicating that Mischka has an acceptable inbreeding level.

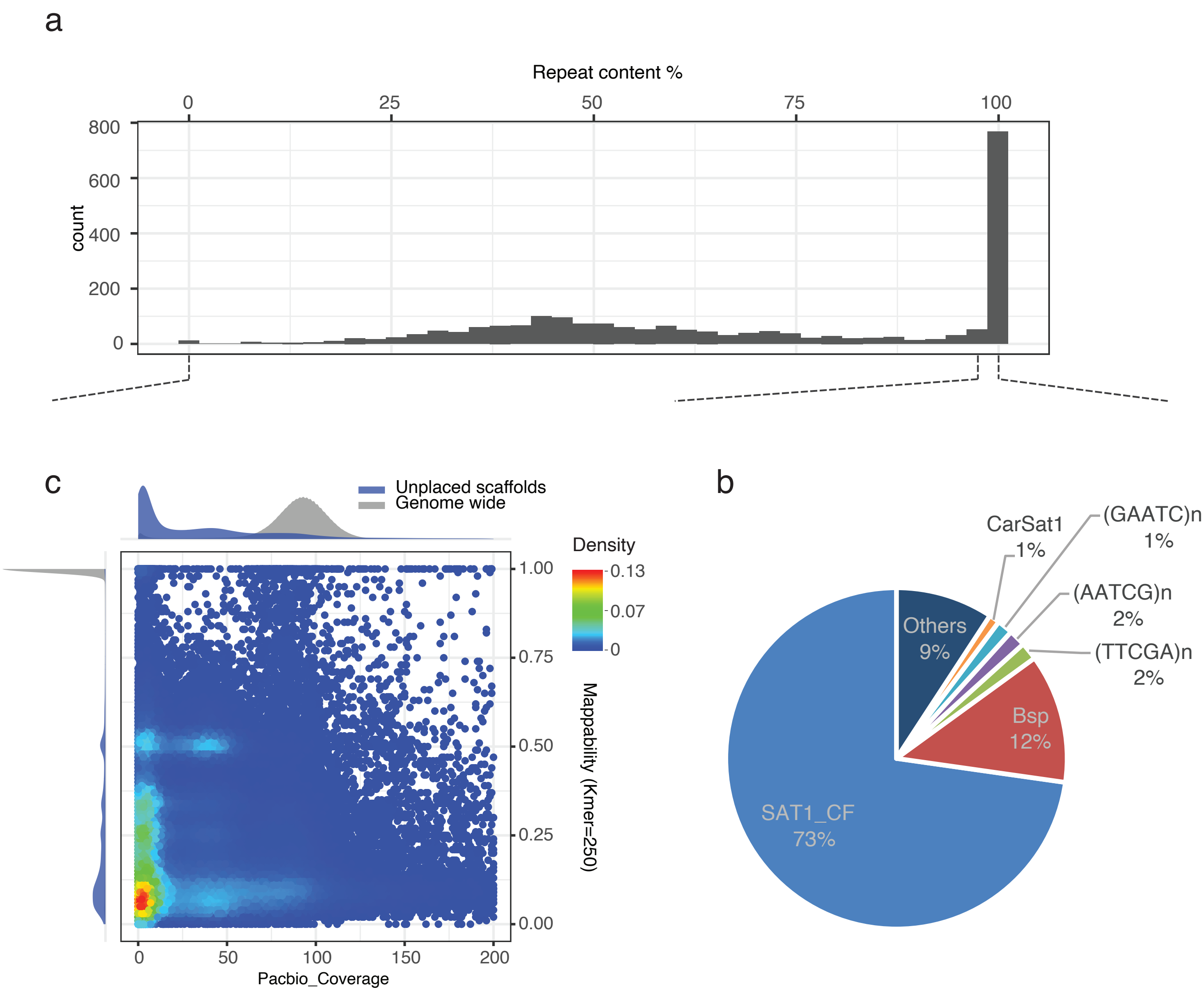

Supplementary Figure 2. Sequence characteristics of 2,159 unplaced scaffolds in GSD_1.0. For each unplaced scaffold, we calculated the repeat content and the average of mappability (k-mer = 250 bp). a) Distribution of the repeat content for unplaced scaffolds. 871 scaffolds are found with content >90%. b) The most common repeat is SAT1_CF, which is a known centromeric repeat. c) Of the remaining scaffolds, most have lower coverage and mappability compared to the chromosome level scaffolds, suggesting they could be from the genomic duplication regions.

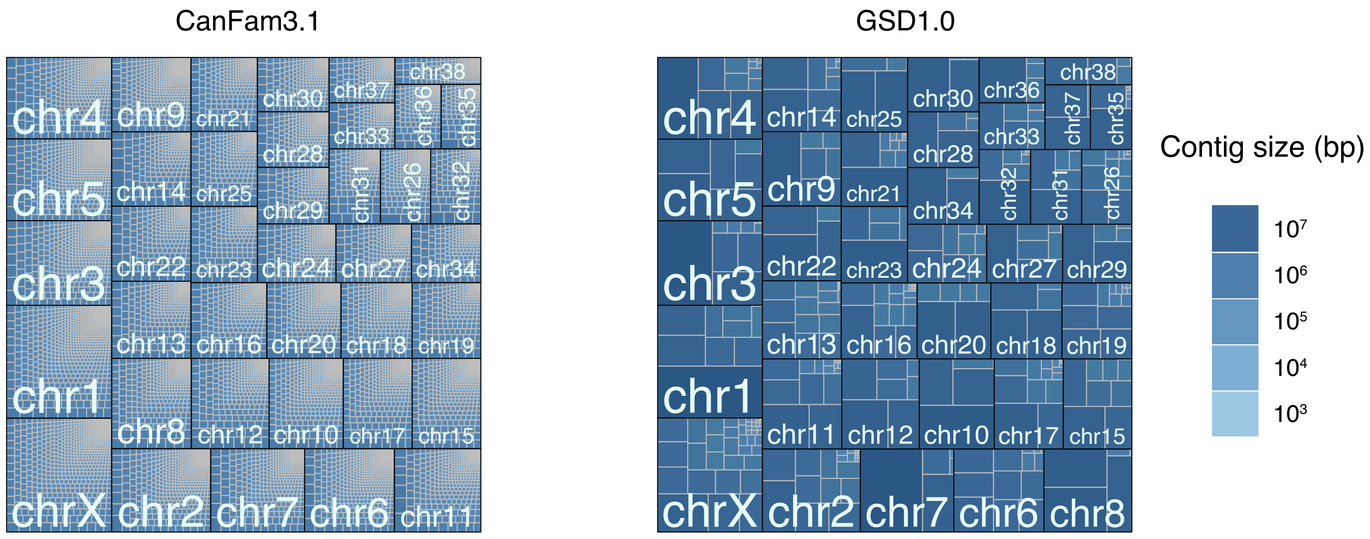

Supplementary Figure 3. Treemap of CanFam3.1 and GSD_1.0 contigs scaled by length.

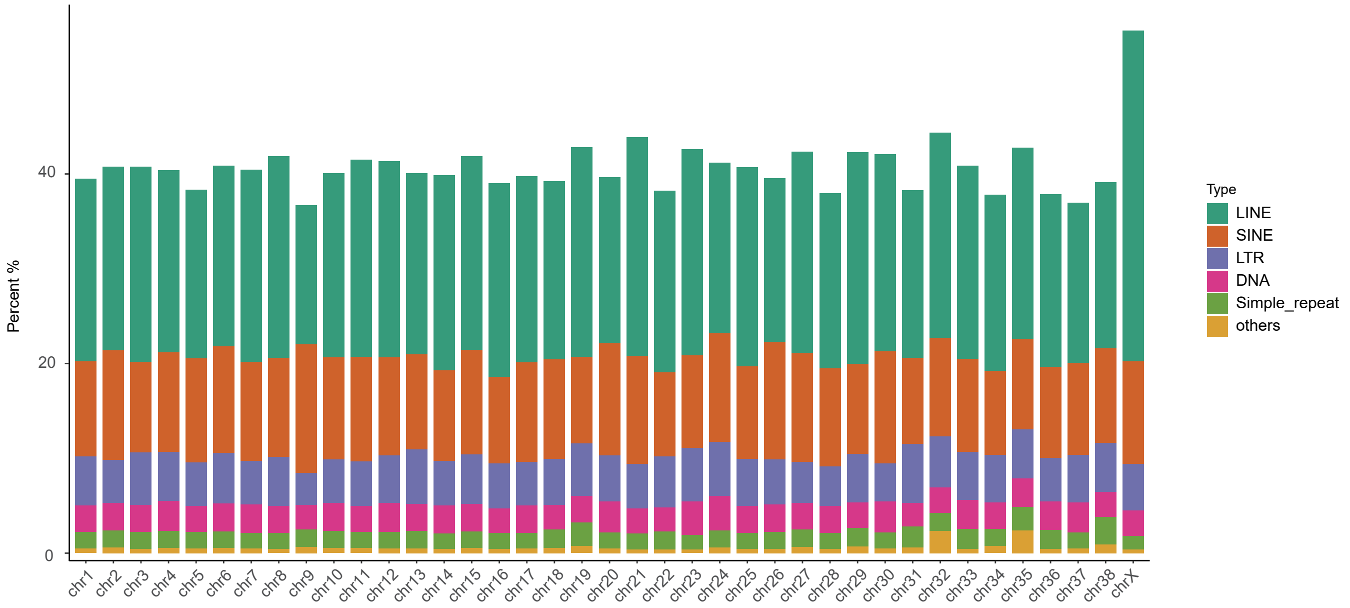

Supplementary Figure 4. Content of repetitive elements on each GSD_1.0 chromosome. The repeat content is stable between autosomes. Chromosome X shows a relatively higher repeat content by virtue of having more LINEs.

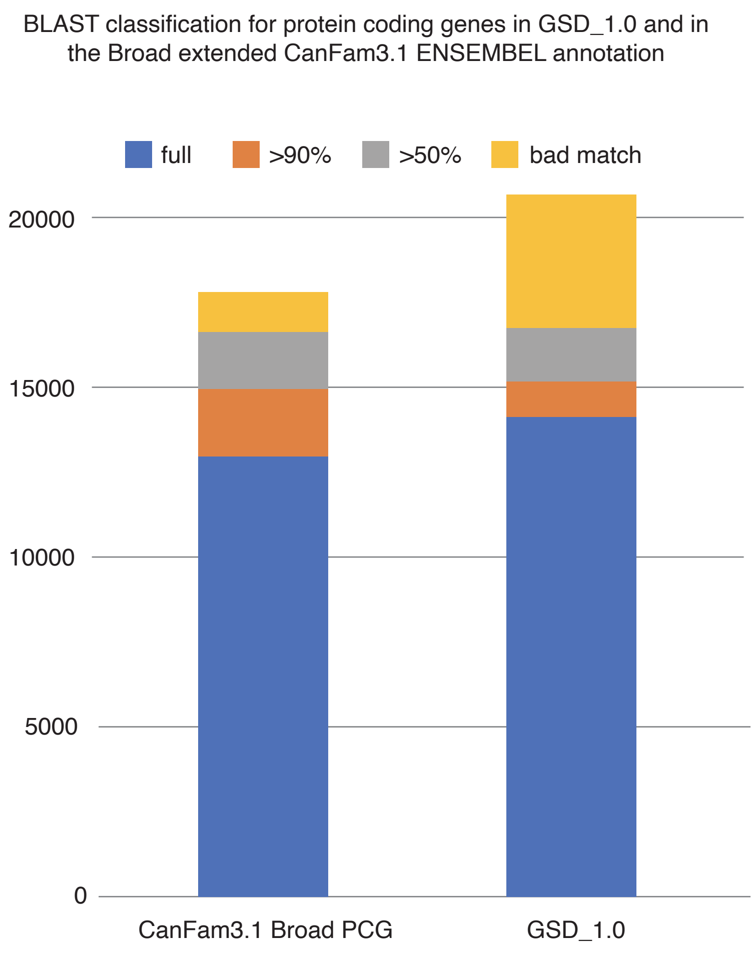

Supplementary Figure 5. Comparison between length of the best BLAST hit for genes in GSD_1.0 and CanFam3.1 annotation.

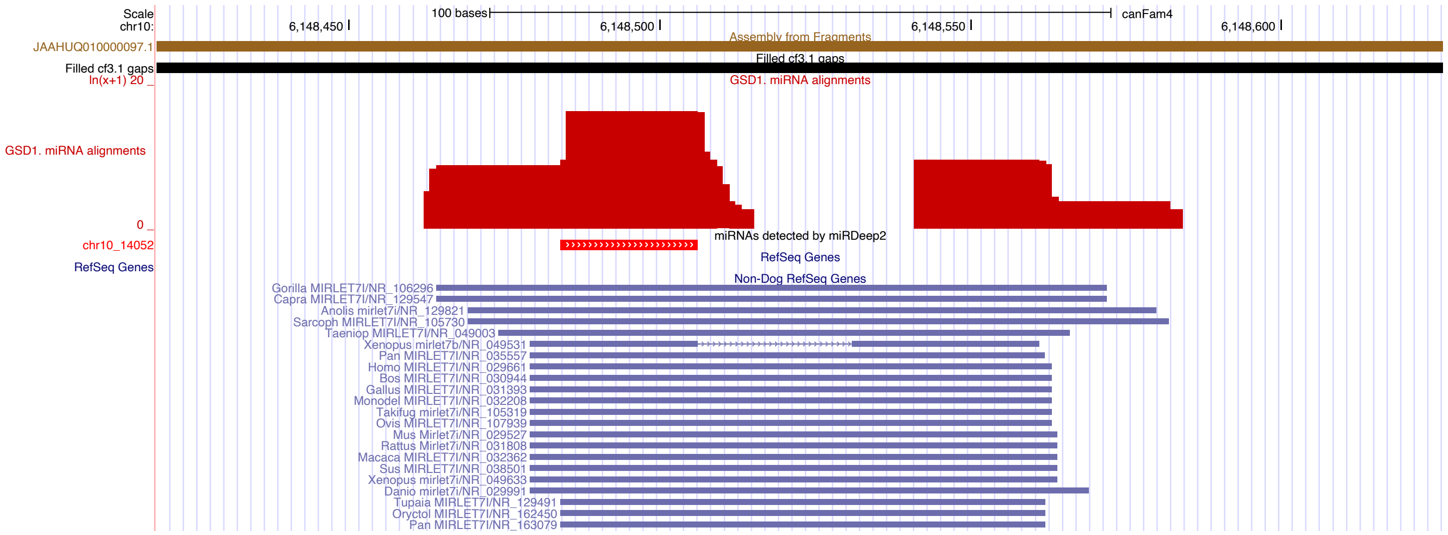

Supplementary Figure 6. Mirlet7i identified from GSD_1.0. mirlet7i has the 16th highest miRDeep2 score, with close to 11 million aligned reads from the combined miRNA-seq dataset located within a filled CanFam3.1 gap.

a

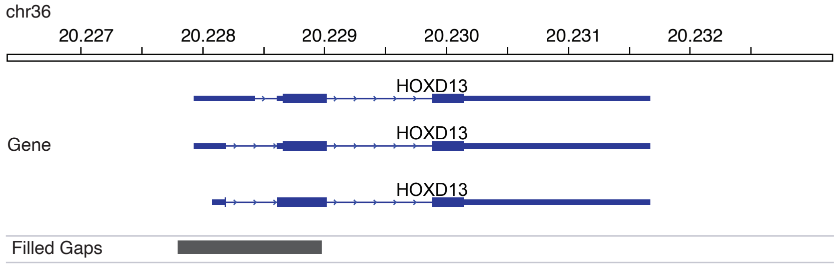

b

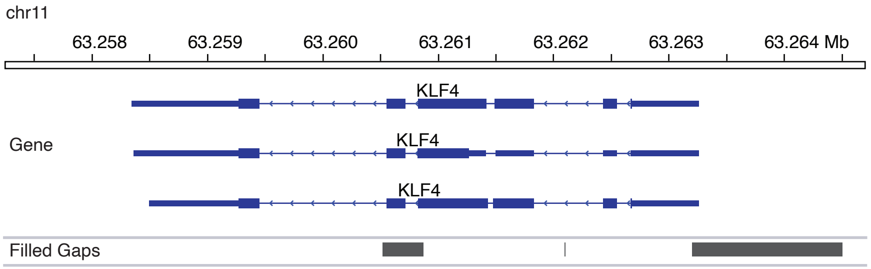

Supplementary Figure 7. Filled CanFam3.1 gaps in cancer genes from the COSMIC database. a) *HOXD13* gene and b) *KLF4* gene

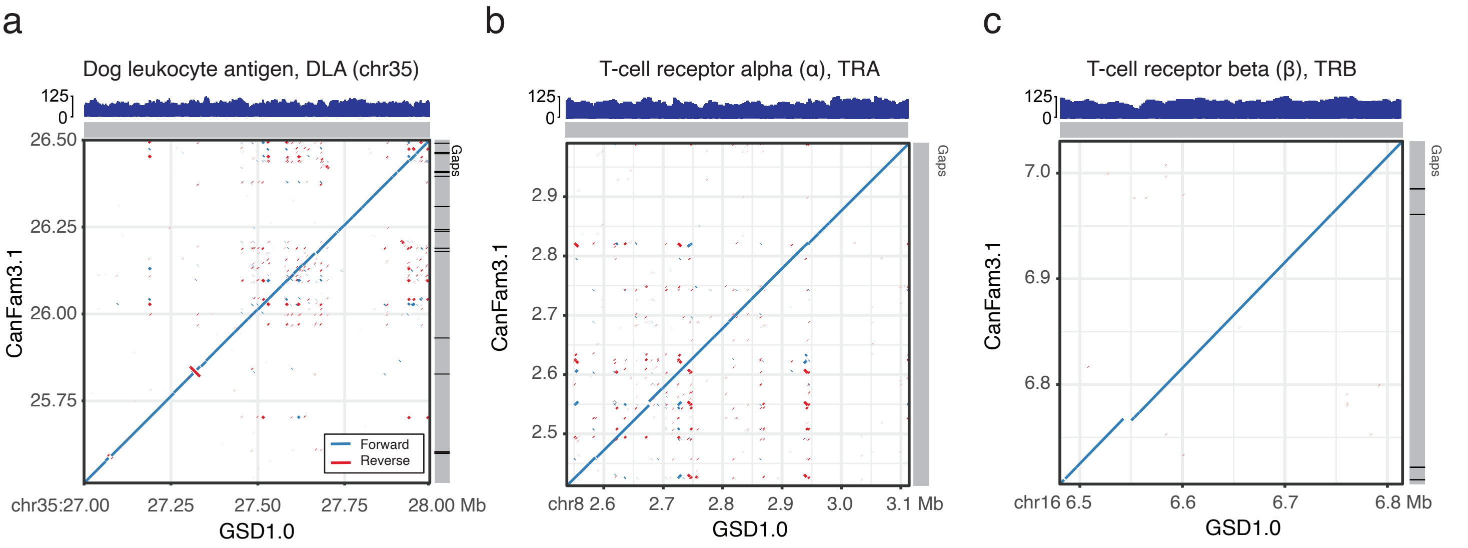

Supplementary Figure 8. Sequence comparison of immunity loci between GSD_1.0 and CanFam3.1. Blue indicates a forward alignment, and red indicates a reverse alignment. A) Dog leukocyte antigen locus (DLA) on chromosome 35. b) The T-cell receptor alpha (TRA) locus on chromosome 8, and c) T-cell receptor beta (TRB) locus on chromosome 16.

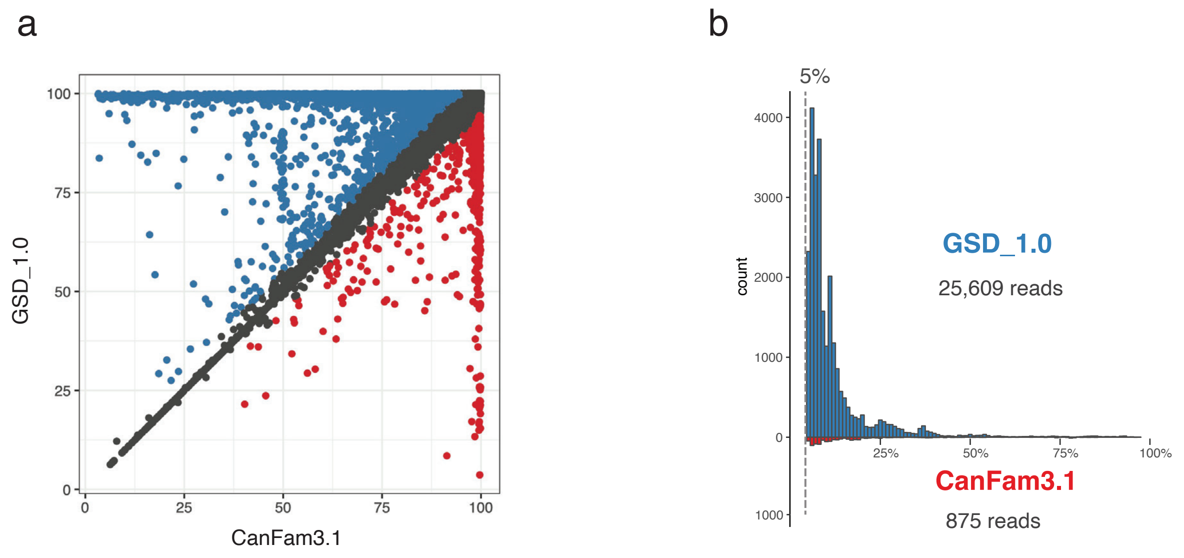

Supplementary Figure 9. Iso-Seq data mapped to GSD_1.0 and CanFam3.1. a) The percentage of mapped bases for each read in the two assemblies. Each dot indicates an Iso-Seq read. Blue dots showed the reads with >5% more bases mapped in the GSD1.0 compared to CanFam3.1. Red dots indicated the reads have 5% more bases mapped in CanFam3.1. b) Distribution of mapping improvement for the two kinds of reads. Altogether 25,609 reads have at least 5% more bases mapped in the GSD_1.0, whereas only 875 reads could be better mapped to CanFam3.1.

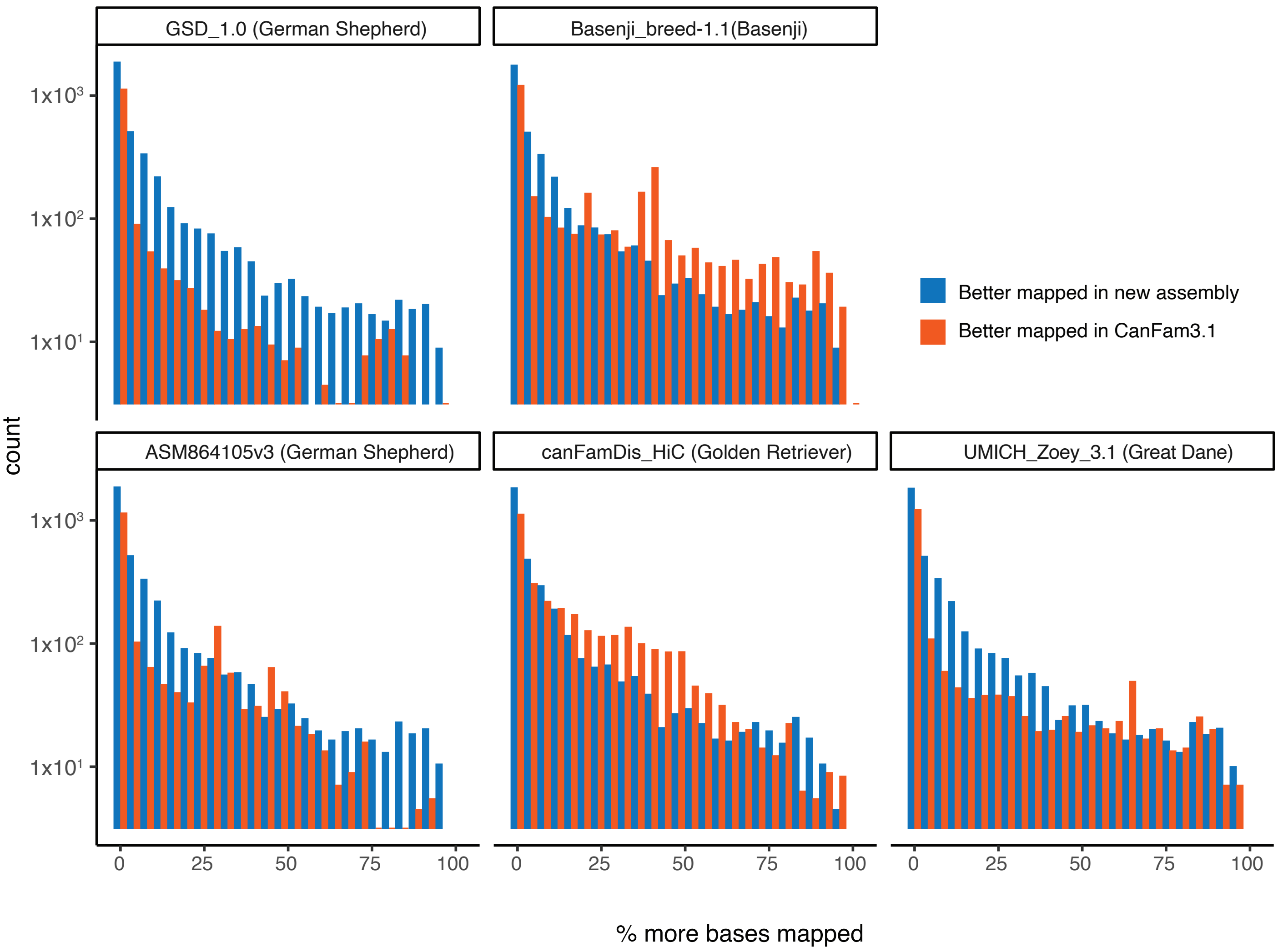

Supplementary Figure 10. Mapping Iso-seq data to different canine assemblies. Comparisons are made to CanFam3.1. We counted the mapped bases of each read, and calculated the difference in terms of percent more bases mapped between two assemblies. A single read could either be better mapped in CanFam3.1 (red) or in the newly released assembly GSD_1.0 (blue)

a

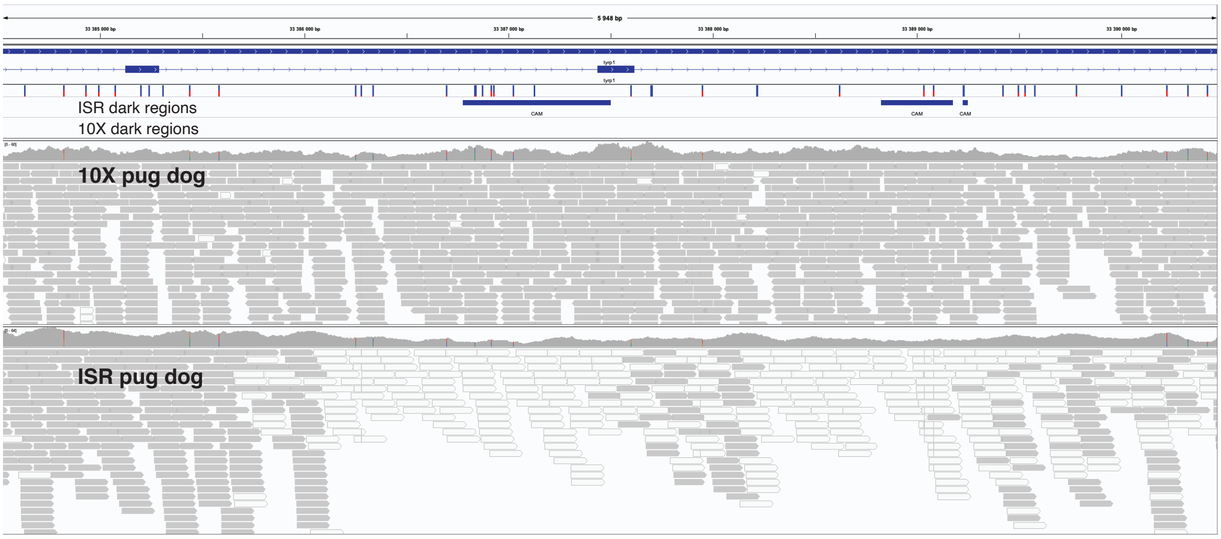

b

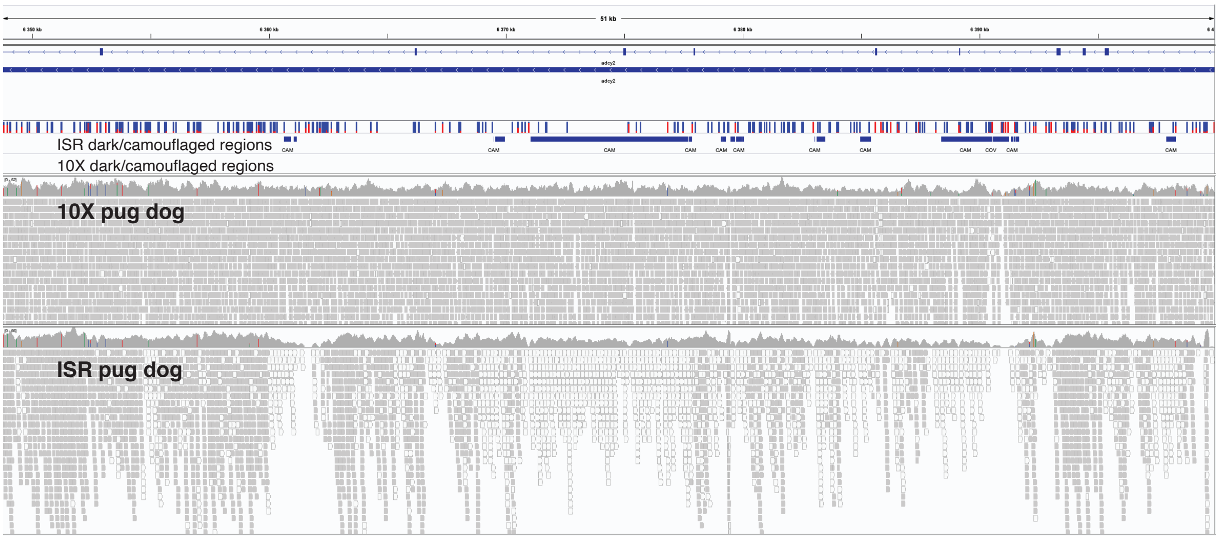

Supplementary Figure 11. Illumina short reads (ISR) dark and camouflaged regions rescued by 10x sequencing. IGV snapshot showed the dark/camouflaged regions detected from the ISR (25 dogs) and 10X (27dogs) datasets, and illustrated the read alignments of one pug dog from each dataset. ISR dark/camouflaged regions in the a) *TYRP1* gene, and b) *ADCY2* gene.

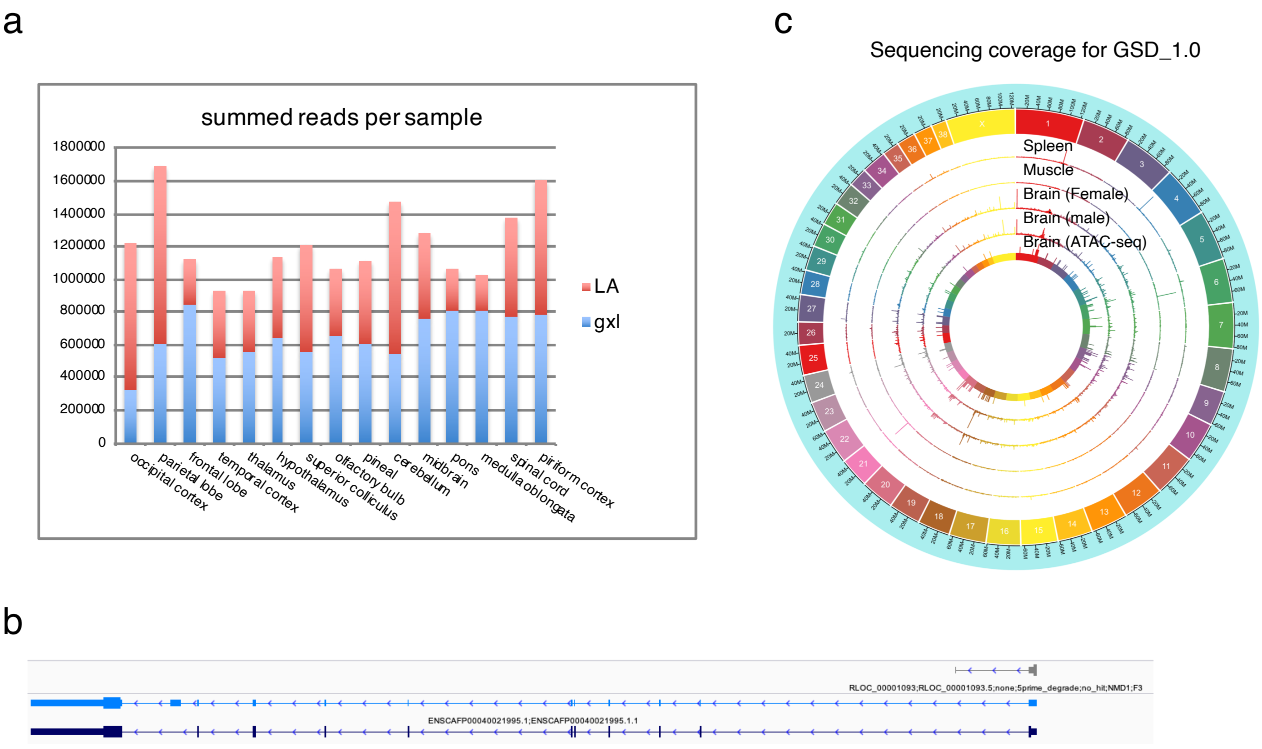

Supplementary Figure 12. RNA sequencing of different tissues. a) The total read numbers per tissue for a multiplexed run (2 flowcells) with female brain samples. N50 of reads was 2.4 kb, and amplification was done with two enzymes (LongAmp and GXL) to avoid coverage bias. b) Annotations were filtered to remove degraded transcripts (gray). Colors indicate the quality of BLAST hit as defined by TAMA: gray = no hit, dark blue = full hit, medium blue = 90% match, light blue = 50% match, light skye blue = bad hit. c) Read distribution for Nanopore samples for various tissues. To avoid issues with high coverage from tissue specific genes in transcript assembly and merging reads were assembled per sample type and resulting transcripts merged.

Supplementary Tables

Supplementary Table 1. Summary of repetitive elements in GSD_1.0

Type<br>subtype	number of elements	length occupied (bp)	percentage of sequence (%)
SINEs	1538330	253126533	10.2
Alu/B1	0	0	0
MIRs	436540	64619318	2.6
LINEs	878624	504530620	20.33
LINE1	534540	413420208	16.66
LINE2	292578	79467264	3.2
L3/CR1	37964	8374531	0.34
RTE	12346	3077052	0.12
LTR element	308696	120262262	4.85
ERVL	90609	40692875	1.64
ERVL-MaLRs	146071	50536666	2.04
ERV_classI	49789	23477942	0.95
ERV_classII	0	0	0
DNA element	325449	68706785	2.77
hAT-Charlie	187859	36590816	1.47
TcMar-Tigger	49675	14764477	0.59
Unclassified	5213	965509	0.04
Total interspersed repeats		947591709	38.18
Small RNA	1141119	191329823	7.71
Satellites	3922	52090599	2.1
Simple repeats	955316	50351697	2.03
Low complexity	129088	7138943	0.29

Supplementary Table 2. Tissues used in RNA experiments

Sample				Purpose*	Tissue RIN																								
ID	Breed	Sex	Age (years)		Cerebellum	Frontal lobe	Hypothalamus	Medulla oblongata	Midbrain	Occipital cortex	Olfactory bulb	Parietal lobe	Pineal	Piriform cortex	Pons	Spinal cord	Superior colliculus	Temporal cortex	Thalamus	Skeletal muscle	Heart	Lung	Pancreas	Kidney	Ovary	Testis	Liver	Spleen	Retina
na§	Beagle	na	na	Iso-Seq	-	-	8.0	-	-	-	-	-	-	-	-	-	-	-	-	-	-	-	-	-	-	-	-	-	-
na	Beagle	Female	na	Nano/miRNA	8.2	8.6	8.8	7.9	7.9	7.6	8.7	8.2	8.4	8.7	8.5	8.6	8.1	7.9	7.9	7.8	8.0	-	-	3.5	5.8	-	-	4.8	-
na	Beagle	Male	na	Nano/miRNA	6.9	9.1	7.7	7.6	6.6	6.3	9.1	7.7	7.2	8.5	8.0	7.2	8.0	8.0	5.8	-	-	7.7	6.2	-	-	5.8	5.0	-	-
na	Beagle	Female	12	Nano/Illumina	-	-	-	-	-	-	-	-	-	-	-	-	-	-	-	-	-	-	-	-	-	-	-	-	8.0
14S	Beagle	Male	na	qCPR	-	-	-	-	-	-	-	-	-	-	-	-	-	-	-	-	-	-	-	-	-	-	6.2	3.6	-
8L/5S	Border Collie	Female	13	qCPR	-	-	-	-	-	-	-	-	-	-	-	-	-	-	-	-	-	-	-	-	-	-	7.8	8.1	-
1S	Cocker Spaniel	Male	9	qCPR	-	-	-	-	-	-	-	-	-	-	-	-	-	-	-	-	-	-	-	-	-	-	8.1	-	-
3S	Dachshund	Female	15	qCPR	-	-	-	-	-	-	-	-	-	-	-	-	-	-	-	-	-	-	-	-	-	-	9.3	-	-
1L/4S	Danish Swedish Farmdog	Female	12	qCPR	-	-	-	-	-	-	-	-	-	-	-	-	-	-	-	-	-	-	-	-	-	-	9.2	7.3	-
10L	German Shepherd	Female	12	qCPR	-	-	-	-	-	-	-	-	-	-	-	-	-	-	-	-	-	-	-	-	-	-	-	8.1	-
18L/12S	German Shepherd	Female	12	qCPR	-	-	-	-	-	-	-	-	-	-	-	-	-	-	-	-	-	-	-	-	-	-	6.0	5.9	-
22L/15S	German Shepherd	Female	5	qCPR	-	-	-	-	-	-	-	-	-	-	-	-	-	-	-	-	-	-	-	-	-	-	7.5	7.6	-
24L/16S	German Shepherd	Female	1	qCPR	-	-	-	-	-	-	-	-	-	-	-	-	-	-	-	-	-	-	-	-	-	-	7.7	8.0	-
6S	German Shepherd	Female	2	qCPR	-	-	-	-	-	-	-	-	-	-	-	-	-	-	-	-	-	-	-	-	-	-	8.5	-	-
25L/17S	German Shepherd	Male	1	qCPR	-	-	-	-	-	-	-	-	-	-	-	-	-	-	-	-	-	-	-	-	-	-	7.4	8.3	-
5L/10S	German Shepherd	Male	12	qCPR	-	-	-	-	-	-	-	-	-	-	-	-	-	-	-	-	-	-	-	-	-	-	7.3	7.6	-
12L/8S	Golden Retriever	Female	11	qCPR	-	-	-	-	-	-	-	-	-	-	-	-	-	-	-	-	-	-	-	-	-	-	7.7	7.7	-
7L	Greyhound	Female	12.5	qCPR	-	-	-	-	-	-	-	-	-	-	-	-	-	-	-	-	-	-	-	-	-	-	-	7.6	-
17L	Hovawart	Female	7	qCPR	-	-	-	-	-	-	-	-	-	-	-	-	-	-	-	-	-	-	-	-	-	-	-	8.6	-
3L/11S	Howawart	Female	11	qCPR	-	-	-	-	-	-	-	-	-	-	-	-	-	-	-	-	-	-	-	-	-	-	8.7	8.0	-
9L	Howawart	Female	9	qCPR	-	-	-	-	-	-	-	-	-	-	-	-	-	-	-	-	-	-	-	-	-	-	-	6.0	-
4L	Howawart	Male	11	qCPR	-	-	-	-	-	-	-	-	-	-	-	-	-	-	-	-	-	-	-	-	-	-	-	6.8	-
11L	Irish Setter	Male	6	qCPR	-	-	-	-	-	-	-	-	-	-	-	-	-	-	-	-	-	-	-	-	-	-	-	7.7	-
6L/2S	Irish Setter	Male	na	qCPR	-	-	-	-	-	-	-	-	-	-	-	-	-	-	-	-	-	-	-	-	-	-	8.8	7.9	-
2L	Mixed Breed	na	na	qCPR	-	-	-	-	-	-	-	-	-	-	-	-	-	-	-	-	-	-	-	-	-	-	-	7.2	-
13L/7S	Poodle Medium	Female	13	qCPR	-	-	-	-	-	-	-	-	-	-	-	-	-	-	-	-	-	-	-	-	-	-	6.6	7.2	-
23L	Swedish Elkhound	Female	9	qCPR	-	-	-	-	-	-	-	-	-	-	-	-	-	-	-	-	-	-	-	-	-	-	-	6.5	-

*Iso-Seq, PacBio Iso-Seq; Nano, nanopore sequencing; miRNA, miRNA-Sequencing; Illumina, Illumina NovaSeq
§ Sample commercially supplied as total RNA
na, not available

Supplementary Table 3. Filled CanFam3.1 gaps within the cancer genes from COSMIC

	COSMIC tier1 genes	COSMIC tier2 genes
Number of genes in GSD1.0	532	128
Number of genes with filled gaps	286	80
Number of the filled gaps	531	182
Exons (count/length)	328/92.2 Kb	94/30.4 Kb
coding sequences (count/length)	241/46.2 Kb	77/18.0 Kb
5'UTR (count/length)	310/61.5 Kb	115/21.0 Kb
3'UTR (count/length)	54/17.7 Kb	14/2.8 Kb

Supplementary Table 4. Summary of available dog assemblies from public resources

Assembly	Origin	Name	Sequencing Tech.	total length (bp)	Number of scaffolds	N50 (bp)	NCBI Accession/Links
CanFam3.1	Boxer	Tasha	BAC+Sanger sequencing	2,410,976,875	3,310	45,876,610	GCF_000002285.3
CanFam GSD1.0	German Shepherd	Mischka	Pacbio+10X+HiC	2,482,000,080	2,198	64,299,765	GCA_011100685.1
ASM864105v3	German Shepherd	Nala	Pacbio+BioNano+HiC	2,407,242,830	410	64,346,267	GCA_008641055.3
UMICH_Zoey_3.1	Great Dane	Zoey	PacBio	2,343,218,756	794	64,204,256	GCA_005444595.1
Basenji_breed-1.1	Basenji	Luka	PacBio	2,410,429,933	2,243	61,087,166	GCA_004886185.2
canFamDis_HiC	Golden Retriever	Scarlet	Illumina+HiC	2,507,649,681	476,756	58,988,005	https://www.dnazoo.org/assemblies/Canis_lupus_familiaris

Supplementary Table 5. ISO-seq reads mapping in different canine assemblies

Assembly	BUSCO score	ISO-seq data			
		N. of mapped reads (change from canFam3.1)	Mapped bases (change from canFam3.1)	N. of reads with >5% mapped based in new assembly (%)	N. of reads with >5% mapped based in canFam3.1 (%)
canFam3.1	95.20%	532,845	1,533,421,214 bp	-	-
CanFam GSD1.0	95.40%	532,959 (+0.02%)	1,543,939,818 bp (+0.69%)	25,609 (4.80%)	875 (0.16%)
ASM864105v3	93.60%	532,949 (+0.02%)	1,543,851,433 bp (+0.68%)	25,408 (4.76%)	4,549 (0.85%)
UMICH_Zoey_3.1	95.20%	532,886 (+0.01%)	1,542,024,196 bp (+0.56%)	25,361 (4.75%)	2,087 (0.39%)
Basenji_breed-1.1	94.80%	532,322 (-0.1%)	1,523,786,336 bp (-0.63%)	25,134 (4.71%)	19,470 (3.65%)
canFamDis_HiC	95.20%	532,930 (+0.02%)	1,528,715,704 bp (-0.31%)	19,988 (3.74%)	23,620 (4.43%)

Supplementary Table 6. 10x sequencing of Mischka and 27 dogs

Dog id	Breed	Sex	Access number	Sequencing depth	Average molecule length
Mischka	German Shepherd	female	SRR10428535	94X	32515 bp
BOX_1	Boxer	male	SRR10441650	48X	49912 bp
CS_1	Cocker Spaniel	female	SRR10441644	48X	58612 bp
FCR_2	Flat-coated Retriever	male	SRR10441645	49X	51482 bp
SCH_1	Schnauzer	male	SRR10441646	36X	50857 bp
BOX_2	Boxer	male	SRR10441649	32X	51860 bp
FCR_1	Flat-coated Retriever	female	SRR10441648	35X	56222 bp
GR_1	Golden Retriever	male	SRR10441647	46X	37071 bp
BM_1	Bernese Mountain	male	SRR10441652	37X	48888 bp
CKCS_1	Cavalier King Charles Spaniel	male	SRR10441637	30X	52694 bp
CS_2	Cocker Spaniel	female	SRR10441651	47X	81696 bp
CS_3	Cocker Spaniel	female	SRR10441643	56X	50670 bp
DH_1	Dachshund	female	SRR10441642	31X	45266 bp
DBM_1	Dobermann	female	SRR10441641	88X	78645 bp
GSD_1	German Shepherd	male	SRR10441640	59X	56919 bp
GSD_2	German Shepherd	female	SRR10441638	39X	63405 bp
GSD_3	German Shepherd	male	SRR10441639	37X	72428 bp
GD_1	Great Dane	female	SRR10441636	43X	69816 bp
IWH_1	Irish Wolfhound	female	SRR10441635	38X	63311 bp
IWH_2	Irish Wolfhound	female	SRR10441634	41X	63563 bp
LEO_1	Leonberger	male	SRR10441633	31X	78586 bp
PUG_1	Pug	male	SRR10441632	30X	76245 bp
RTW_1	Rottweiler	male	SRR10441631	32X	52249 bp
RTW_2	Rottweiler	male	SRR10441630	56X	44930 bp
SS_1	Springer Spaniel	female	SRR10441629	43X	61677 bp
SBT_1	Staffordshire Bull Terrier	male	SRR10441628	34X	69289 bp
SP_1	Standard Poodle	female	SRR10441627	44X	66798 bp
WM_1	Weimaraner	female	SRR10441626	51X	59408 bp

Supplementary Table 7. Gene expression summary for structural variant loci

SV	Locus	Location[2]	Gene	Expression p-value (samples)[1]		
				Liver	Spleen	Heart
Deletion	MANEA	chr12:54,394,997-54,440,487	*MANEA*	0.31 (het=6, del=14)	0.95 (het=5, del=10)	na
Deletion	RAB32	chr1:37,945,215-37,966,816	*RAB32*	0.17 (wt=6, del=3)	na (wt=6, del=2)	na
Deletion	POLI	chr1:21,276,172-21,301,190	*POLI*	na	na	0.95 (wt=3,het=3,del=3)
CNV	OTOA	chr6:23,345,645-23,406,849	*OTOA*	na (CNV2=5; CNV>2=2)	na (CNV2=5; CNV>2=1)	na
			IGSF6	na (CNV2=5; CNV>2=2)	na (CNV2=5; CNV>2=1)	na
			METTL9	na (CNV2=5; CNV>2=2)	na (CNV2=5; CNV>2=1)	na
CNV	PPHLN1	chr27:34,959,082-35,101,842	*PPHLN1*	0.64 (CNV2=13; CNV>2=7)	0.54 (CNV2=8; CNV>2=7)	na
			YAF	0.49 (CNV2=13; CNV>2=7)	0.73 (CNV2=4; CNV>2=4)	na
			ZCRB1	0.13 (CNV2=13; CNV>2=7)	0.614 (CNV2=8; CNV>2=7)	na
CNV	CYP1A2	chr30:38,258,389-38,264,108	*CYP1A2*	0.66 (CNV3=7; CNV>3=11)	0.90 (CNV3=5; CNV>3=9)	na
			CLK3	0.28 (CNV3=7; CNV>3=8)	0.28 (CNV3=5; CNV>3=8)	na
			CSK	0.54 (CNV3=7; CNV>3=9)	0.61 (CNV3=5; CNV>3=9)	na
			SCAMP2	0.45 (CNV3=6; CNV>3=7)	0.11 (CNV3=4; CNV>3=4)	na
			ULK3	na (CNV3=2; CNV>3=2)	0.11 (CNV3=2; CNV>3=6)	na

1 Samples available per locus per genotype; SV, structural variant type; na, not applicable.

2 Location with GSD1.0 co-ordinates. For PPHLN1 and CYP1A2, the break point was determined from reads, not PCR validation

Supplementary Table 8. Sequencing data generated from three HiC libraries

library	Number of read pairs	Bases count (bp)	Coverage	Accession
hic1	118,370,222	35,747,807,044	14.30X	SRR10428538
hic2	133,709,087	40,380,144,274	16.15X	SRR10428537
hic3	150,143,152	45,343,231,904	18.14X	SRR10428536

Supplementary Table 9. Public resource of illumina short-read data of 25 dogs

Sample ID	Number of paired reads	Reads length (bp)	Sequencing platform	Accession	Genome coverage	Breed
ISR_BOX_1	285156478	125	Illumina HiSeq 2500	ERR2196023	28X	Boxer
ISR_CKCS_1	276885105	125	Illumina HiSeq 2500	ERR2196025	28X	Cavalier King Charles Spaniel
ISR_DH_1	364316971	150	Illumina HiSeq 2000	ERR3047535	43X	Dachshund
ISR_PUG_1	344029880	150	Illumina HiSeq 2000	ERR3047544	41X	Pug
ISR_LEO_1	203589257	100	Illumina HiSeq 3000	SRR7107523	16X	Leonberger
ISR_CS_1	222108500	150	Illumina HiSeq 2500	SRR7107632	26X	Cocker spaniel
ISR_WM_1	192335020	150	Illumina HiSeq 2500	SRR7107634	23X	Weimaraner
ISR_GSD_1	194141819	100	Illumina HiSeq 2000	SRR7107763	16X	German Shepherd
ISR_GSD_2	188797150	100	Illumina HiSeq 2000	SRR7107767	15X	German Shepherd
ISR_GSD_3	194166764	100	Illumina HiSeq 2000	SRR7107769	16X	German Shepherd
ISR_CS_2	176178243	100	Illumina HiSeq 2000	SRR7107863	14X	English cocker spaniel
ISR_SCH_1	218290866	100	Illumina HiSeq 2000	SRR7107866	17X	Standard Schnauzer
ISR_SS_1	406968239	100	Illumina HiSeq 2000	SRR7107883	31X	English Springer Spaniel
ISR_SP_1	276357019	100	Illumina HiSeq 2000	SRR7107900	22X	Standard Poodle
ISR_DBM_1	372622195	100	Illumina HiSeq 2000	SRR7107901	29X	Doberman
ISR_GR_1	376930517	100	Illumina HiSeq 2000	SRR7107926	29X	Golden retriever
ISR_GD_1	250616106	100	Illumina HiSeq 2500	SRR7107941	20X	Great Dane
ISR_BM_1	473640347	125	Illumina HiSeq 2500	SRR7120127	47X	Bernese Mountain Dog
ISR_FCR_1	410367234	125	Illumina HiSeq 2500	SRR7120158	41X	Flat-coated retriever
ISR_RTW_1	241991725	150	Illumina HiSeq 2500	SRR7120208	22X	Rottweiler
ISR_RTW_2	502519042	125	Illumina HiSeq 2500	SRR7120209	50X	Rottweiler
ISR_BOX_2	320189252	100	Illumina HiSeq 2000	SRR8541911	23X	Boxer
ISR_IWH_1	370755703	100	Illumina HiSeq 2000	SRR8541930	24X	Irish Wolfhound
ISR_IWH_2	377090701	100	Illumina HiSeq 2000	SRR8541931	24X	Irish Wolfhound
ISR_CS_3	208440846	100	Illumina HiSeq 2500	SRR8614081	16X	English cocker spaniel

Supplementary References

1. Tengvall, K. et al. Genome-Wide Analysis in German Shepherd Dogs Reveals Association of a Locus on CFA 27 with Atopic Dermatitis. PLOS Genet. 9, e1003475 (2013).
