# Supplementary material for: A novel canine reference genome resolves genomic architecture and uncovers transcript complexity
Source: Commun Biol. 2021 Feb 10;4:185. doi: 10.1038/s42003-021-01698-x (PMC7875987; doi:10.1038/s42003-021-01698-x)
Supplement: Supplementary file 2 — Description of Additional Supplementary Files [file 42003_2021_1698_MOESM2_ESM.pdf]

Description of Additional Supplementary Files

File Name: Supplementary Data 1
Description: Datasets used for annotation

File Name: Supplementary Data 2
Description: Primers used for validation and genotyping

File Name: Supplementary Data 3
Description: Dark and camouflaged regions detected in each dog

File Name: Supplementary Data 4
Description: Tissues samples and genotyping results for expression analysis
