# Supplementary material for: A novel canine reference genome resolves genomic architecture and uncovers transcript complexity
Source: Commun Biol. 2021 Feb 10;4:185. doi: 10.1038/s42003-021-01698-x (PMC7875987; doi:10.1038/s42003-021-01698-x)
Supplement: Supplementary file 7 — Reporting summary [file 42003_2021_1698_MOESM7_ESM.pdf]

nature research

Corresponding author(s): Chao Wang
Kerstin Lindblad-Toh

Last updated by author(s): Aug 17, 2020

Reporting Summary

Nature Research wishes to improve the reproducibility of the work that we publish. This form provides structure for consistency and transparency in reporting. For further information on Nature Research policies, see our Editorial Policies and the Editorial Policy Checklist.

Statistics

For all statistical analyses, confirm that the following items are present in the figure legend, table legend, main text, or Methods section.

n/a	Confirmed	
☐	☒	The exact sample size (n) for each experimental group/condition, given as a discrete number and unit of measurement
☐	☒	A statement on whether measurements were taken from distinct samples or whether the same sample was measured repeatedly
☐	☒	The statistical test(s) used AND whether they are one- or two-sided<br>*Only common tests should be described solely by name; describe more complex techniques in the Methods section.*
☒	☐	A description of all covariates tested
☒	☐	A description of any assumptions or corrections, such as tests of normality and adjustment for multiple comparisons
☐	☒	A full description of the statistical parameters including central tendency (e.g. means) or other basic estimates (e.g. regression coefficient) AND variation (e.g. standard deviation) or associated estimates of uncertainty (e.g. confidence intervals)
☐	☒	For null hypothesis testing, the test statistic (e.g. F, t, r) with confidence intervals, effect sizes, degrees of freedom and P value noted<br>*Give P values as exact values whenever suitable.*
☒	☐	For Bayesian analysis, information on the choice of priors and Markov chain Monte Carlo settings
☒	☐	For hierarchical and complex designs, identification of the appropriate level for tests and full reporting of outcomes
☐	☒	Estimates of effect sizes (e.g. Cohen's d, Pearson's r), indicating how they were calculated

Our web collection on statistics for biologists contains articles on many of the points above.

Software and code

Policy information about availability of computer code

Data collection	No software was used for data collection.
Data analysis	All scripts used for data analysis were deposited to https://github.com/Chao912/Mischka/

For manuscripts utilizing custom algorithms or software that are central to the research but not yet described in published literature, software must be made available to editors and reviewers. We strongly encourage code deposition in a community repository (e.g. GitHub). See the Nature Research guidelines for submitting code & software for further information.

Data

Policy information about availability of data

All manuscripts must include a data availability statement. This statement should provide the following information, where applicable:

- Accession codes, unique identifiers, or web links for publicly available datasets
- A list of figures that have associated raw data
- A description of any restrictions on data availability

The PacBio-long reads, HiC, and Illumina 10x data of Mischka are available in SRA under BioProject PRJNA587469. RNA, miRNA and ATAC-seq data used for annotation are available in SRA under BioProject PRJNA657719. The Illumina 10x data of 27 dogs are available in SRA under BioProject PRJNA588624. The canFam_GSD_1.0 assembly is deposited in DDBJ/ENA/GenBank under JAAHUQ000000000.

Field-specific reporting

Please select the one below that is the best fit for your research. If you are not sure, read the appropriate sections before making your selection.

☒ Life sciences ☐ Behavioural & social sciences ☐ Ecological, evolutionary & environmental sciences

For a reference copy of the document with all sections, see nature.com/documents/nr-reporting-summary-flat.pdf

Life sciences study design

All studies must disclose on these points even when the disclosure is negative.

Sample size	The size of samples used for 10x and RNA-seq were determined to fully represent the variations across dogs breeds and tissues.
Data exclusions	Low quality Reads from DNA, RNA or ATAC-seq were excluded from the analysis.
Replication	All results and findings from this study are reproducible.
Randomization	This is not relevant to this genome assembly study.
Blinding	This is not relevant to this genome assembly study.

Reporting for specific materials, systems and methods

We require information from authors about some types of materials, experimental systems and methods used in many studies. Here, indicate whether each material, system or method listed is relevant to your study. If you are not sure if a list item applies to your research, read the appropriate section before selecting a response.

Materials & experimental systems

n/a	Involved in the study	
☒	☐	Antibodies
☒	☐	Eukaryotic cell lines
☒	☐	Palaeontology and archaeology
☐	☒	Animals and other organisms
☒	☐	Human research participants
☒	☐	Clinical data
☒	☐	Dual use research of concern

Methods

n/a	Involved in the study	
☒	☐	ChIP-seq
☒	☐	Flow cytometry
☒	☐	MRI-based neuroimaging

Animals and other organisms

Policy information about studies involving animals; ARRIVE guidelines recommended for reporting animal research

Laboratory animals	This study did not involve laboratory animals.
Wild animals	This study did not involve wild animals.
Field-collected samples	This study did not involve samples collected from the field.
Ethics oversight	Approval was obtained from dog owners before collecting the biological samples at veterinary clinics. Ethical approvals for sampling were granted by Uppsala Animal Ethical Committee and Swedish Board of Agriculture (C139/9, C2/12, C12/15). Importation of canine tissues was approved by Jordbruksverket (6.7.18-14513/17).

Note that full information on the approval of the study protocol must also be provided in the manuscript.
